# CryoEM structure of the tegumented capsid of Epstein-Barr virus

**DOI:** 10.1038/s41422-020-0363-0

**Published:** 2020-07-03

**Authors:** Zhihai Li, Xiao Zhang, Lili Dong, Jingjing Pang, Miao Xu, Qian Zhong, Mu-Sheng Zeng, Xuekui Yu

**Affiliations:** 1 Cryo-Electron Microscopy Research Center, The CAS Key Laboratory of Receptor Research, Shanghai Institute of Materia Medica, Chinese Academy of Sciences, Shanghai, 201203 China; 2 State Key Laboratory of Oncology in South China, Collaborative Innovation Center for Cancer Medicine, Guangdong Key Laboratory of Nasopharyngeal Carcinoma Diagnosis and Therapy, Department of Experimental Research, Sun Yat-sen University Cancer Center, Sun Yat-sen University, Guangzhou, Guangdong 510060 China; 3 University of Chinese Academy of Sciences, Beijing, 100049 China

**Keywords:** Cryoelectron microscopy, Mechanisms of disease

## Abstract

Epstein-Barr virus (EBV) is the primary cause of infectious mononucleosis and has been shown to be closely associated with various malignancies. Here, we present a complete atomic model of EBV, including the icosahedral capsid, the dodecameric portal and the capsid-associated tegument complex (CATC). Our in situ portal from the tegumented capsid adopts a closed conformation with its channel valve holding the terminal viral DNA and with its crown region firmly engaged by three layers of ring-like dsDNA, which, together with the penton flexibility, effectively alleviates the capsid inner pressure placed on the portal cap. In contrast, the CATCs, through binding to the flexible penton vertices in a stoichiometric manner, accurately increase the inner capsid pressure to facilitate the pressure-driven genome delivery. Together, our results provide important insights into the mechanism by which the EBV capsid, portal, packaged genome and the CATCs coordinately achieve a pressure balance to simultaneously benefit both viral genome retention and ejection.

## Introduction

There are seven known human oncogenic viruses, which account for about 15%–20% of all human cancers.^1^ Epstein-Bar virus (EBV), a member of the subfamily gammaherpesviruses that also includes the Kaposi Sarcoma-associated herpesvirus (KSHV),^2,3^ is the first identified oncovirus.^4,5^ EBV infects over 90% of the population worldwide^6^ and has been shown to be closely associated with various malignancies, including Hodgkin’s lymphoma,^7^ Burkitt’s lymphoma,^3^ NK/T cell lymphoma,^8^ nasopharyngeal carcinoma^9^ and gastric carcinoma.^10^

Similar to other members of the family herpesviridae, EBV has a characteristic three-layer configuration: the outer lipid bilayer envelope containing viral glycoproteins responsible for host recognizing and membrane fusion, the inner pseudo-icosahedral nucleocapsid enclosing a 172-kb double-strand DNA (dsDNA) genome and the middle pleomorphic tegument compartment fulfilled with 20–40 different viral proteins.^11–14^

All herpesvirus capsid shell consists of 4 abundant proteins: the major capsid proteins (MCP) constituting the basic capsid skeleton through forming 150 hexons and 11 pentons, the small capsid proteins (SCP) binding to the tops of hexon MCPs or both hexon and penton MCPs, and the Tri1 and Tri2 forming the heterotrimeric triplexes to plug the large holes on the capsid floor.^15–18^ Once the viral dsDNA genome enters the procapsid shell through the portal that is located at a unique 5-fold vertex of the icosahedral capsid, the viral nucleocapsid is joined by the capsid-associated tegument complexes (CATCs).^19^ The composition and binding pattern of CATCs vary among different herpesviruses, which is likely in order to cope with the different inner pressures resulted from the packaged genomes that have sizes ranging from 125 to 235 kb.^20^ For example, while the human cytomegalovirus (HCMV) capsid contains the largest genome of 235-kb dsDNA and has both the pentons and hexons being secured by CMV-specific pp150 tegument protein,^15,21^ the herpes simplex virus (HSV) and KSHV containing much smaller genomes of 150 kb and 165 kb, respectively, have only their pentons being secured by CATCs.^17,18,22^

With the rapid advance of cryo-electron microscopy (cryoEM), the atomic structures of several herpesviruses, such as HCMV, HSV-1, HSV-2, and KSHV,^15–18^ have been determined, and more recently, the in situ portal structures of HSV-1 and KSHV were also resolved by cryoEM.^22,23^ In contrast to these great successes, the progress toward an atomic description of the EBV capsid has been hindered, likely by the difficulties of EBV virion sample preparation, and the highest-resolution reconstruction of the EBV capsid, which was determined 8 years ago, remains 20 Å.^24^ Here, by using an optimized viral culture method modified from previous procedure^25^ and cryoEM, we obtained high-resolution reconstructions of the EBV icosahedral capsid, dodecameric portal and CATC. The genome size-related flexibility of capsid pentons, the closed portal conformation, the unique portal-DNA engagement and stoichiometric binding of CATCs observed in our tegumented capsid structure enabled us to suggest new functional roles of the capsid penton vertices, portal, and CATCs, which in turn provides insights into the events related to viral genome packaging, retention and ejection.

## Results

### Structures of the capsid, in situ portal and CATCs

By using the new viral growth method, we obtained a high-quality EBV sample preparation (Supplementary information, Fig. S1a). To increase the signal-to-noise ratio of the images, we added detergent into the purified intact virus sample immediately before grid freezing to break down the viral envelope in order to obtain the tegumented nucleocapsid (Supplementary information, Fig. S1b).

**Fig. 1:**
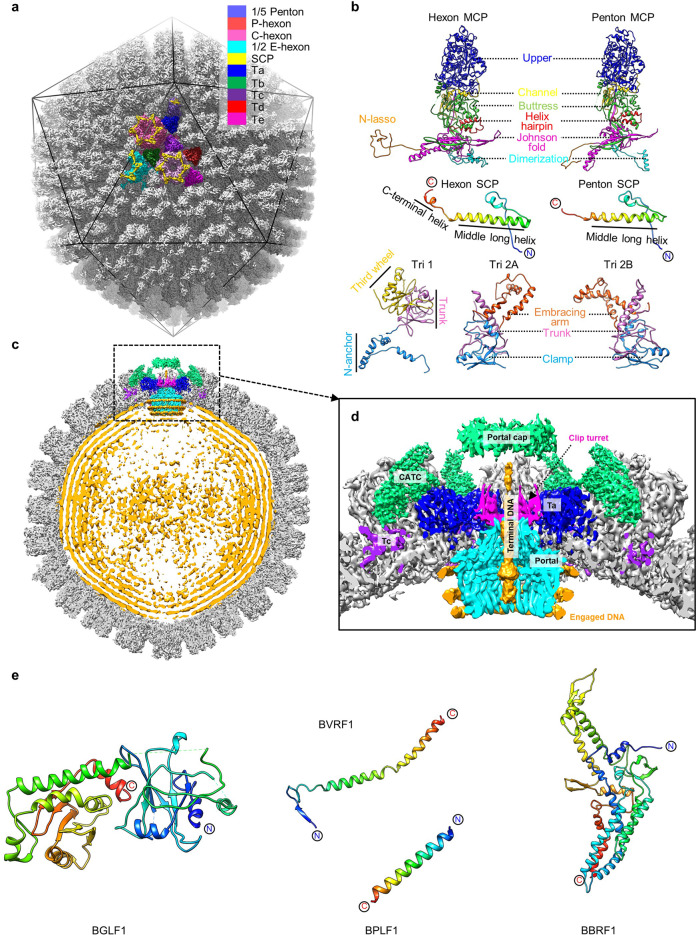
Structures of the EBV capsid, in situ portal and CATC. **a** The icosahedral reconstruction of EBV capsid. One asymmetric unit is colored by molecule. **b** Models of the capsid proteins. The SCP is rainbow colored by residue. All the other proteins are colored by domains. **c** Central slice of the capsid asymmetric reconstruction, showing the portal vertex and the packaged dsDNA. **d** Zoomed-in view of the boxed region in **c**, showing the interactions among portal, capsid proteins and viral genome DNA. **e** Models of CATC proteins. Each protein is rainbow colored by residue.

We obtained an icosahedral reconstruction at a resolution of 4.1 Å from 32,721 particles (Fig. 1a; Supplementary information, Fig. S2 and Table S1), which, complemented by a 3.5 Å structure of the penton-vertex region through sub-particle classification and refinement (Supplementary information, Fig. S2 and Table S1), allowed us to build an ab initio atomic model for the viral capsid, including 16 copies of MCPs, 16 copies of SCPs, 5 copies of the triplex monomer proteins (Tri1) and 10 copies of triplex dimer proteins (Tri2) (Fig. 1a, b; Supplementary information, Figs. S3–S5). By symmetry relaxation and localized classification,^23^ we sequentially resolved the structures of the C5 portal vertex, the C12 portal, the C1 portal vertex and the asymmetric capsid at resolutions of 4.2 Å, 4.8 Å, 5.5 Å and 7.5 Å, respectively (Fig. 1c, d; Supplementary information, Figs. S2 and S3 and Movie S1), which enable us to build atomic models for the portal protein BBRF1 and the CATC proteins of BVRF1, BPLF1 and BGLF (Fig. 1e; Supplementary information, Fig. S6 and Tables S1, S2). The asymmetric capsid structure showed the following: (1) the dodecameric portal interacts with five copies of the peripenton hexon (P-hexon) and triplexes of Ta in a 12–5-fold symmetry-mismatch pattern; (2) the terminal viral genome DNA is held by the portal channel valve and is capped by a featureless density called portal cap, named after the counterparts in HSV-1 and KSHV^22,23^ (Fig. 1d); and (3) three layers of ring-like viral genome densities tightly wind the portal (Fig. 1d).

### Atomic models and interactions of viral capsid proteins

The MCP molecule can be thought of as an assembly of a tower and a floor. The tower region, which makes up the capsomer protrusions, is composed of the upper (a.a. 487–1043), channel (a.a. 411–486 and 1334–1381), buttress (a.a. 1119–1333) and helix-hairpin (a.a. 190–239) domains. The floor region is composed of dimerization (a.a. 298–375), N-lasso (a.a. 1–61), and characteristic Johnson-fold domain (a.a. 62–189, 240–297, 376–410, and 1044–1118)^26^ (Fig. 1b).

**Fig. 2:**
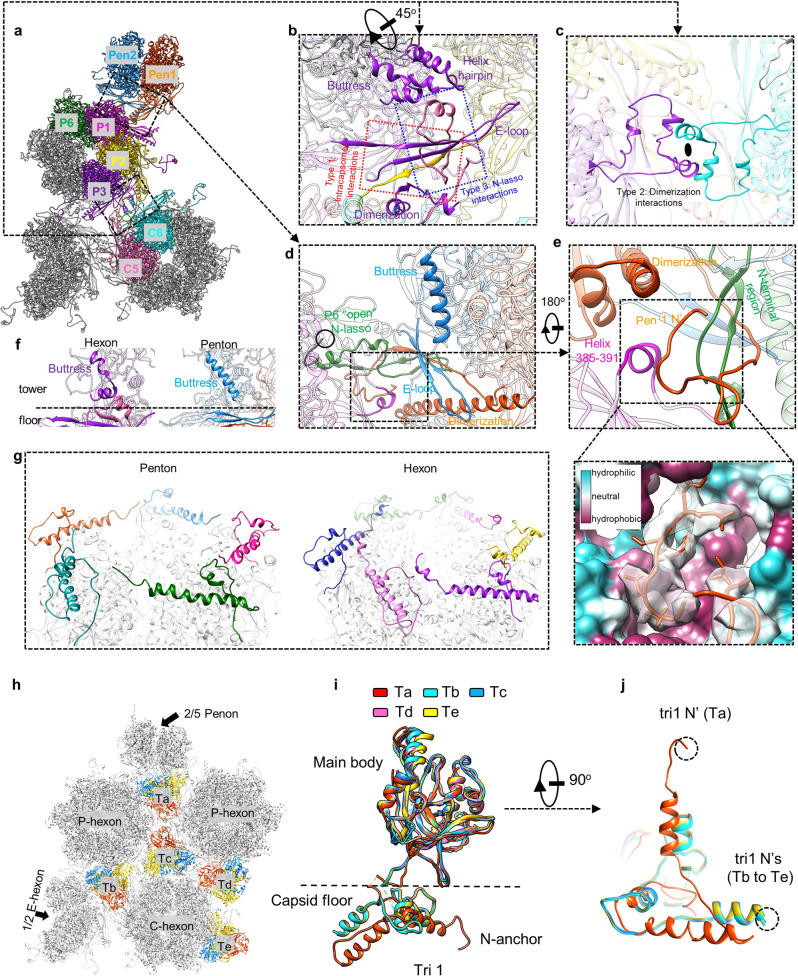
Interactions among capsid proteins. **a** Overview of the MCP interaction network. Two penton MCPs (Pen1 and Pen2) are displayed. **b**, **c** Three types of MCP-MCP interactions among hexons. **d** Interactions between penton MCPs and hexon MCPs. **e** Enlarged view of the boxed region in **d**, showing the N-terminus of the penton MCP inserting into a hydrophobic groove (inset). The hydrophobic residues are highlighted by side chain showing (inset). **f** Comparison of buttress domains of hexon and penton MCPs. The MCP tower and floor regions are labeled, respectively. **g** The MCP-SCP interactions in pentons and hexons. **h** Overview of triplexes and MCPs. **i**, **j** Superimposition of triplexes Ta-Te by aligning their main bodies, showing the Tri1 N-anchor of Ta rotated ~120° counterclockwise.

As observed in other herpesviruses,^15–18,27^ the EBV MCPs are extensively interconnected through three types of interactions between the floor regions of MCPs. The type 1 interaction is an intracapsomer β-augmentation between two neighboring MCPs within a hexon or penton capsomer (Fig. 2a, b). Two β-strands in the E-loop of the Johnson-fold domain and two β-strands in the dimerization domain of P3 are augmented by one β-strand from the N-lasso domain of P2 to form a five-stranded β-sheet. The type 2 interaction is an intercapsomer and quasiequivalent interaction between two pairs of α-helices from the dimerization domains of MCPs across local 2-fold axes (Fig. 2a, c). The type 3 interaction is built upon and further strengthens the type 1 interaction. The N-lasso domain from one MCP subunit extends outward with its two β-strands to augment the five-stranded β-sheet formed through the type 1 interaction of P2 and P3 to produce a seven-stranded β-sheet, which is then lashed by the N-lasso of C5. In addition, this N-lasso is further secured through its helix, which is held in place by a helix bundle of 4 short helices contributed by the P3’s helix-hairpin and buttress domains (Fig. 2b).

The conformation and the network interactions of the penton MCP underwent several changes from those of the canonical hexon MCP described above. First, the dimerization domain of a penton MCP contains only a long helix and is unable to form type 2 interactions with the dimerization domain of a P1 MCP (Fig. 2d). Second, the N-terminus of a penton MCP is partially flexible and, rather than forming a lasso, adopts a compact loop configuration contacting a hydrophobic surface formed by its dimerization domain, a short helix in the Johnson-fold domain of the P1 MCP and the N-terminal region of the P6 MCP (Fig. 2e). Meanwhile, the P6 MCP N-lasso adopts an “open” configuration that eliminates its ability to lash a penton MCP (Fig. 2d). Therefore, a penton MCP neither lashes a hexon MCP nor is lashed by a hexon MCP. Finally, the elbow-like helix-turn-helix structure in the buttress domain of a hexon MCP is instead a long straight helix in a penton MCP and directly contacts the MCP floor region to support the MCP tower (Fig. 2f).

Each of the EBV SCPs binds the tops of two adjacent MCPs both in a hexon and in the penton. In a hexon, one SCP bridges two MCPs through two helices: the middle long helix (a.a. 36–60) binds the groove of one MCP, and a C-terminal short helix binds a neighboring MCP. In a penton, however, the corresponding region of the C-terminal short helix is disordered, with the remaining 3-residue loop contacting the MCP (Fig. 2g).

The triplexes, heterotrimers of one Tri1 molecule and two Tri2 conformers (Tri2A and Tri2B), plug the large holes on the capsid floor (Fig. 2h; Supplementary information, Fig. S7a). Each Tri2 conformer consists of a clamp (a.a. 1–89), a trunk (a.a. 90–192 and a.a. 282–301) and an embracing arm domain (a.a. 193–281) (Fig. 1b; Supplementary information, Fig. S7b, c). The clamp and trunk domains are essentially identical between Tri2A and Tri2B, whereas their embracing arms exhibit significantly different configurations to facilitate the two Tri2 conformers to “embrace” each other (Supplementary information, Fig. S7b, c). The Tri1 protein also consists of three domains: N-anchor (a.a. 1–87), trunk (a.a. 88–228), and third-wheel (a.a. 229–364) (Fig. 1b). Through its third-wheel domain binding the two interdigitated embracing arms of Tri2A and Tri2B, Tri1 joins the Tri2 homodimer to form a heterotrimeric triplex (Fig. 1b; Supplementary information, Fig. S7a). The triplex is then anchored to the capsid by the N-anchor of Tri1 that traverses the capsid floor, extends along the capsid inner surface and folds into a tripod of helices (Fig. 2h–j; Supplementary information, Fig. S7d–g).

There are five types of triplexes, Ta to Te, located at local 3-fold axes. While each of the triplexes Tb to Te interacts with three hexon MCPs, the triplex Ta contacts both P-hexon MCPs (two) and a penton MCP (Fig. 2h). The triplexes Tb to Te adopt a similar structure, whereas the Ta has a unique conformation with its N-anchor rotated ~120° counterclockwise compared to the others (Fig. 2i, j). Accordingly, the N-anchor and capsid floor interactions are different between Ta and the other four triplexes. While the N-anchor of each of the triplexes Tb to Te binds to three hydrophobic surfaces formed by a long helix and its associated β-sheet in the Johnson-fold domains from three hexon MCPs arranged around the local 3-fold axis, the N-anchor of Ta interacts with three hydrophobic surfaces formed exclusively by helices in the Johnson-fold domains and the dimerization domains from two hexon MCPs and two penton MCPs (Supplementary information, Fig. S7d–g). It is worth noting that the main body of the triplex Ta has obviously lower resolution and worse density quality than the counterparts from the other 4 triplexes (Supplementary information, Figs. S3d and S8). This discrepancy results from the fact that the 60 Ta molecules adopt two distinct conformations: the main body of one Ta conformer is rotated ~120° compared to that of another Ta conformer for CATC binding, which will be discussed in detail later.

### Structure of the EBV portal and its interactions with capsid proteins

**Fig. 3:**
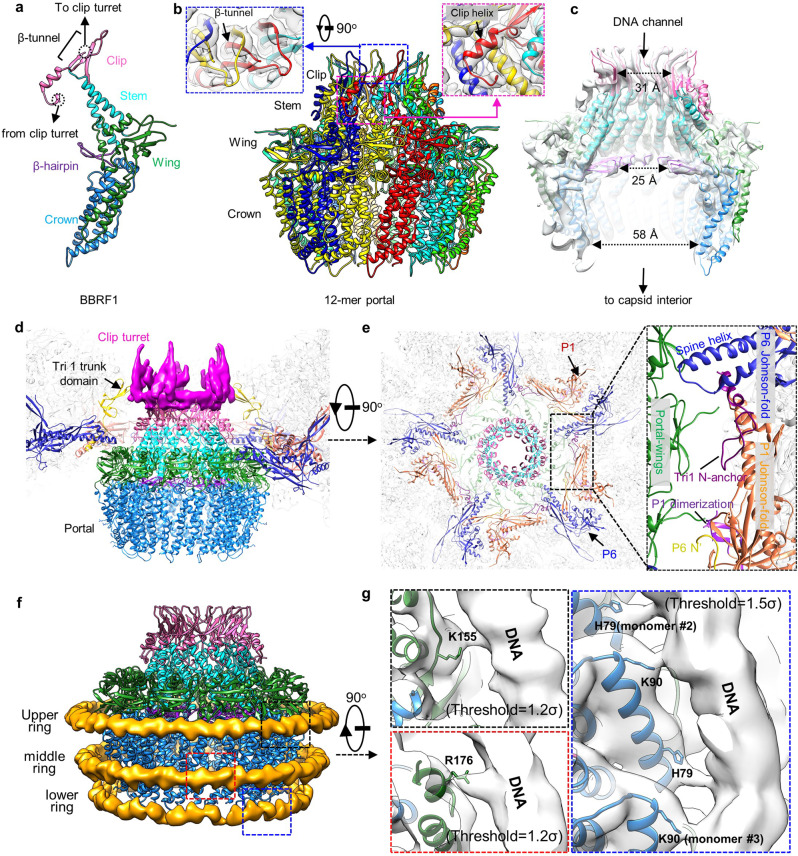
Portal structure and its interactions with capsid proteins and genome DNA. **a** BBRF1 model, colored by domain. **b** The C12 portal structure colored by molecule. Insets, density map (transparent gray) and atomic model of the boxed regions, showing the β augmentation (blue box) and the clip helix holding two neighboring monomers (magenta box). **c** A cutaway view of the portal, showing its interior structure. **d**, **e** Side (**d**) and top (**e**) views of the portal vertex, showing the interaction between portal and capsid proteins. The clip turret density is segmented from the C5 reconstruction of the portal vertex. Inset in **e** shows the secondary elements of capsid proteins involved in the interactions with the portal wing. **f**, **g** Interactions between the portal and the genome DNA. **f** Model of the portal (colored as in **a**) and density map of dsDNA encircling the portal. **g** Zoomed-in views of the boxed regions in **f**, showing the side chains of basic residues of BBRF1 that are involved in the interactions with DNA.

Based on the density map of the C12 portal reconstruction, we built an atomic model of the portal protein BBRF1 (Fig. 3a). The BBRF1 consists of five domains, including wing (a.a. 16–53, 133–160 and 203–252), crown (a.a. 54–132,161–202 and 516–577), stem (a.a. 253–280 and 474–497), clip (a.a. 281–473, including an unmodeled region (a.a. 285–432)), and β-hairpin (a.a. 498–515) (Fig. 3a). Similar to that in KSHV,^22^ we also observed a turret-like density located between the C12 clip domain and the portal cap (Fig. 1d). Based on the agreement between the turret structure that is rich in helix-like densities and the secondary structure prediction of BBRF1 (Supplementary information, Fig. S9), we tentatively assigned the turret to the unmodeled region of BBRF1’s clip domain.

The in situ EBV portal, a dodecamer of BBRF1, shows a mushroom-like shape that is similar to that of the in vitro-assembled EBV portal^28^ (Fig. 3b). The portal channel has two narrow regions that interact directly with the terminal DNA (Figs. 1d and 3c). The first one, located at the interface between the stem and crown domains, is the channel valve formed by 12 copies of β-hairpins protruding toward the channel axis. The channel valve is the most constricted region of the portal channel, with a diameter of ~25 Å (Fig. 3c). Similar to the counterparts in tailed bacteriophages, the channel valve of the EBV portal should play key roles in DNA retention and ejection.^29–31^ The second narrow region is a tunnel-like structure composed of 12 sets of three-stranded β-sheets radially arranged around the channel axis, with a diameter of 31 Å (Fig. 3b, c). Each β-sheet is formed through β-augmentation between a β-strand from the clip domain of one BBRF1 monomer and a two-parallel-stranded β-sheet from the clip base of its counterclockwise neighbor (viewing toward the capsid interior). In addition to β-augmentation, the dodecamer portal is further stabilized by the clip helix (a.a. 440–450) of each BBRF1 monomer, which extends toward the lower left to simultaneously hold the stem regions of its two neighbors (Fig. 3b). The above two characteristic intermonomer interactions not only stabilize the dodecameric portal structure but also may facilitate the timely and coordinately conformational transformation between the dwell and burst states during DNA packaging, as observed in the tailed bacteriophage φ29.^32^

By docking the atomic models of the periportal capsid proteins and dodecameric portal into the C1 reconstruction of the portal vertex, we identified two portal regions involved in interactions with capsid proteins (Fig. 3d, e). The first one is the clip turret, which interacts with the trunk domains of five sets of Ta tri1 molecules (Fig. 3d). The second region is the portal wing, which interacts with capsid floor elements, including five sets of the spine helix in the P6 Johnson-fold, the β-barrel in the P1 Johnson-fold and the N-anchor of the periportal Ta (Fig. 3e).

### Interactions between the EBV portal and viral genome DNA

Both the C1 capsid and the C12 portal reconstructions reveal that the crown region of the portal is encircled by three layers of ring-like DNA densities (Supplementary information, Fig. S10a, b and Movie S1), in stark contrast with intact KSHV^22^ and HSV-1^23^ virions, which have their portals wound by only one layer of DNA density (Supplementary information, Fig. S10b). The C1 reconstruction of the portal vertex clearly shows connecting densities between the portal and each of the three layers of ringed DNA (Supplementary information, Fig. S11). The portal-DNA interactions are contributed mainly by four basic residues (H79, K90, R155 and R176) of BBRF1 (Fig. 3f, g). Compared to the in vitro-assembled EBV portal,^28^ our in situ portal exhibited a more compact conformation with gradually decreased interior diameters from the clip region (31 Å vs 31 Å) downward through the channel valve (25 Å vs 32 Å) to the crown region (58 Å vs 75 Å) (Supplementary information, Fig. S12 and Movie S1).

### The CATC organization on the EBV capsid

**Fig. 4:**
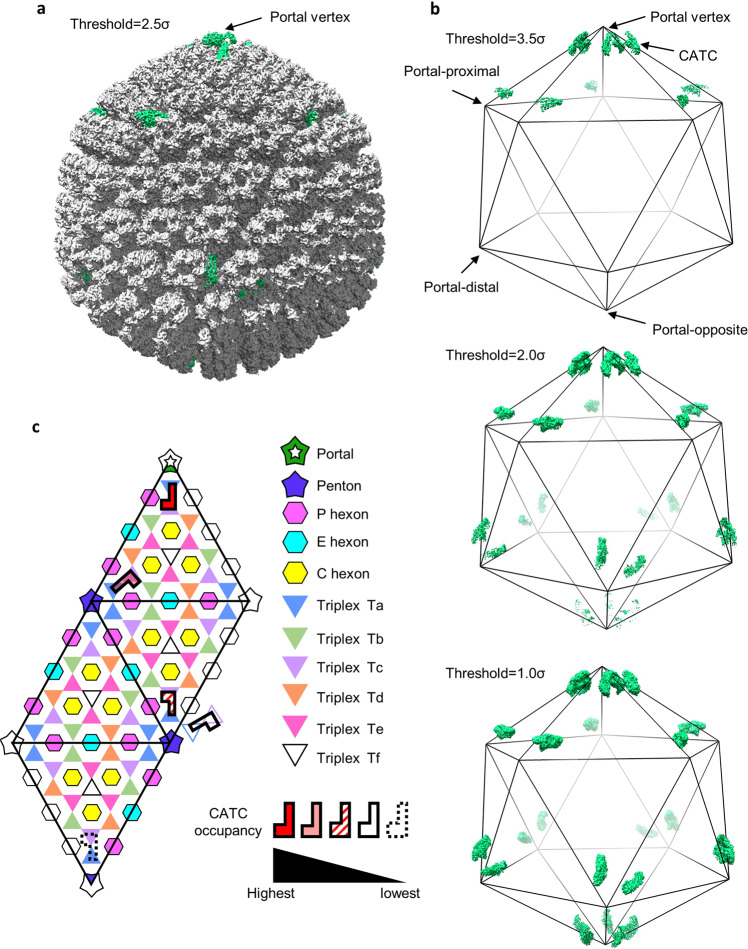
CATC organization on the EBV capsid. **a** C1 capsid reconstruction. The CATCs are highlighted in green. **b** C1 capsid reconstruction displayed at different contour levels. For clarity, the capsid protein and the head domains of BVRF1 proteins are removed. **c** A schematic of one asymmetric unit (shaded) of the capsid with a pseudo-5-fold symmetry, illustrating the CATC occupancy on vertex registers of EBV capsid.

The C1 reconstruction of our portal vertex revealed CATC densities are as strong as those of the surrounding capsid proteins, indicating full occupancy of the five CATC registers surrounding the portal (Fig. 1d). In contrast, each of the CATC registers around the 11 penton vertices is either lacking a CATC or has low occupancy, as indicated by the lower density strength than that at the portal vertex (Fig. 4a, b). By lowering the display threshold of the C1 capsid density map, we found that the EBV capsid shows gradually decreased CATC occupancies on vertex registers from the portal-proximal penton through the portal-distal penton to the portal-opposite penton (Fig. 4b, c). However, the CATC-binding pattern of the EBV capsid does not follow the “portal-side equatorial rule” that was first revealed in the KSHV capsid.^22^ For example, only one of the two portal-side equatorial registers of the portal-proximal penton in our C1 capsid structure is occupied by a CATC. Similarly, only two of the three equatorial registers of the portal-distal penton could be occupied by a CATC (Fig. 4b).

To analyze the CATC occupancy at the EBV penton vertices, we expanded the dataset of penton-vertex sub-particles with 5-fold symmetry and then performed focused 3D classification with a mask including only one CATC copy (Supplementary information, Fig. S2). We finally obtained one 3D class (20.3%) with CATC density strength comparable to that of the surrounding capsid proteins. Notably, the 20.3% occupancy of the penton-vertex registers in EBV is significantly lower than that (37.9%) in KSHV,^22^ albeit EBV contains a larger DNA genome. We also identified 37.9% of the penton vertices in EBV are CATC-absent and obtained a reconstruction of the C5 CATC-absent penton vertex at 3.8 Å by 3D refinement (Supplementary information, Figs. S2 and S3).

### Structure of the CATC and its interactions with the triplex

**Fig. 5:**
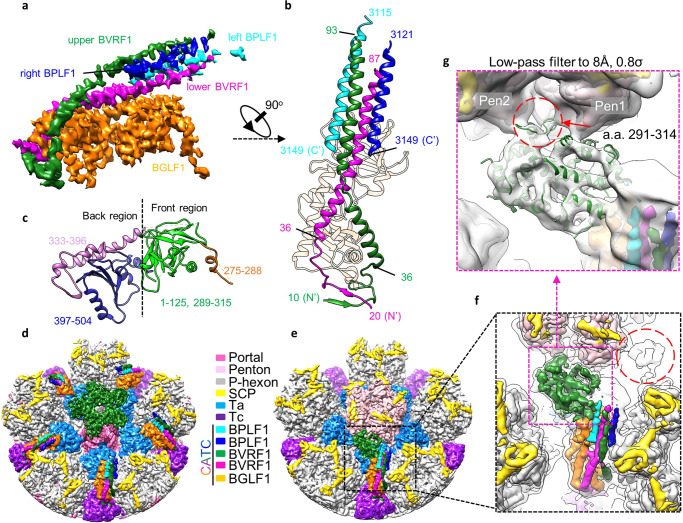
CATC structure. **a** The density map of CATC, colored by its different composition. **b** Top views of the CATC structure. **c** Model of BGLF1, colored by domain. **d**, **e** C1 reconstructions of the portal (**d**) and portal-proximal penton (**e**) vertices. **f** Zoomed-in view of the boxed region in **e**, which is superimposed with an 8 Å low-pass-filtered map (transparent gray). The two head domains of the two BVRF1 conformers are indicated by magenta box and red circle, respectively. **g** Zoomed-in view of **f**, showing the interaction between the head domain of one BVRF1 conformer and two penton MCPs.

Based on the 4.2 Å density map of the C5 portal vertex, we built an atomic model of the CATC, including 65% of full-length BGLF1 (331 of 507 residues), two copies of the BVRF1 N-terminal region (a.a. 10–93 for the upper one and a.a. 20–87 for the lower one) and two copies of the BPLF1 C-terminal region (a.a. 3115–3149 for the left one and a.a. 3121–3149 for the right one) (Fig. 5a–c). We also determined the high-resolution structure of the CATC in portal-proximal penton vertices where the CATC binds exclusively to a specific one of the five registers (Fig. 4b). In order to do so, we performed focused 3D classification of the portal-proximal penton-vertex sub-particles (extracted from the C1 capsid) with a mask encompassing a region containing only one CATC. We finally obtained 93,334 (83.3%) CATC-binding portal-proximal penton vertex sub-particles (Supplementary information, Fig. S2). Through 3D refinement, we obtained reconstruction of the C1 CATC-binding portal-proximal penton vertex at 4.3 Å (Supplementary information, Fig. S3). The structure of CATC in the portal-proximal penton vertex is essentially identical to that in the portal vertex (Fig. 5d, e).

Each of the two BVRF1 conformers contains an N-terminal domain (a.a. 10–36 for the upper one and a.a. 20–36 for the lower one) and a helix domain (a.a. 37–93 for the upper one and a.a. 37–87 for the lower one) (Fig. 5b). The helix domains of the BVRF1 molecules and the two BPLF1 C-terminal regions form a helix bundle lying on the BGLF1 molecule. We observed two globular densities at two sides of the CATC helix bundle, with the one on the left showing stronger intensity than the one on the right (Fig. 5f). We assigned the two globular densities to the head domains of the two BVRF1 molecules. Indeed, the atomic model of the HSV-1 pUL25 (BVRF1 homolog) head domain (PDB: 2F5U)^33^ could reliably fit into the left-side globular density, which shows clear secondary structure elements in the 8 Å low-pass filtered map. We then built a homology model of the BVRF1 head domain by using the model of the pUL25 head domain as a reference (Fig. 5f). Unlike its counterpart in KSHV that interacts with only one MCP,^22^ the head domain of BVRF1 simultaneously contacts two neighboring penton MCPs, mainly through the region of residues 291–314 (Fig. 5g).

The BGLF1 protein can be divided into two regions: the front region, including the N-terminal β-strand-rich domain (a.a. 1–125 and a.a. 289–315) and the triplex Ta anchor domain (a.a. 275–288), and the back region, including the C-terminal β-strand-rich domain (a.a. 397–504) and the central helix-rich domain (a.a. 333–396) (Fig. 5c). BGLF1 is tightly associated with the top helix bundle mainly through β-augmentation: one β-strand from the helix-rich domain of BGLF1 (a.a. 371–373) is joined by two strands from the N-terminal domains of both the upper (a.a. 12–14) and the lower (a.a. 23–25) BVRF1 proteins (Fig. 5b).

**Fig. 6:**
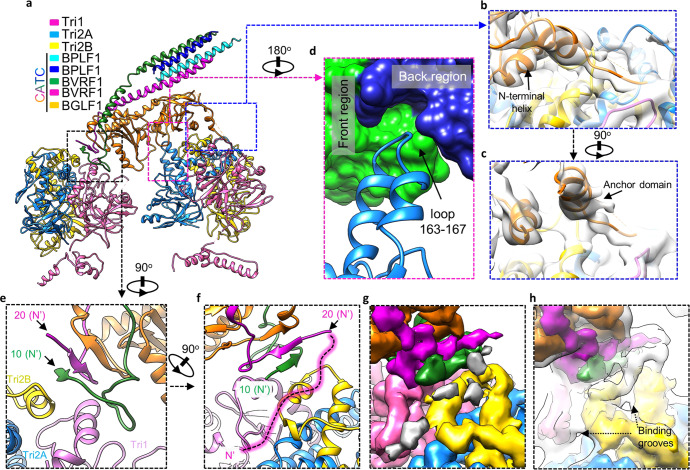
Interactions between the CATC and underlying triplexes. **a** Ribbon models of a CATC and triplexes Ta and Tc. **b**, **c** Density (transparent gray) and model (ribbon) of the blue boxed region in **a**, demonstrating the interactions between the anchor domain and N-terminal helix of BGLF1 and Ta. **d** Zoomed-in view of the magenta boxed region in **a**, illustrating that a loop in Tri2A of Ta inserts into a shallow groove of BGLF1 (surface representation). **e**–**h** Ribbon models and densities of the black boxed region in **a**, illustrating the CATC-Tc interactions. The dashed line in **f** illustrates the tracing of the unmodeled N-terminal region of the BVRF1 protein. **g** Density map of the same region as in **f**, showing the discontinues densities putatively contributed by the unmodeled N-terminal region of the BVRF1. **h** Superimposition between the sharpened map (colored as in **g**) and the unsharpened map (transparent gray), showing the discontinued densities in the sharpened map became continued densities in the unsharpened map and are connected to the N-terminal extension of the upper BVRF1.

Each CATC binds both triplexes Ta and Tc. The interactions between the CATC and Ta are exclusively mediated by BGLF1. The N-terminal helix (a.a. 1–10) and the anchor domain of BGLF1 contact the surface of triplex Ta (Fig. 6a–c); a shallow groove formed by the four-stranded β-barrel in the front region and two helices in the back region accommodates a short loop (a.a. 163–167) connecting two helices from the Tri2A molecule (Fig. 6a, d). Intriguingly, compared to that of the CATC-binding triplex Ta, the main body of the CATC-absent triplex Ta is rotated ~120°, whereas their N-anchor regions essentially remain the same (Fig. 6e; Supplementary information, Fig. S13a), which is also the case in KSHV.^22^ However, in KSHV, only the main body of CATC-binding Ta rotates ~120° compared to those of triplexes Tb-Te (Supplementary information, Fig. S13b). In contrast, the main bodies of both the CATC-absent and CATC-binding Ta in EBV exhibit ~120° rotations counterclockwise and clockwise, respectively (Supplementary information, Fig. S13a). This finding indicates that the difference in conformation between the CATC-absent and CATC-binding Ta in EBV is not induced by CATC binding, but instead is predetermined, probably by the local curvature of the capsid floor as well as interactions with the surrounding MCPs.^34^

The interactions between the CATC and triplex Tc occur at the vertex-distal end of the CATC (Fig. 6a). The N-terminal loop of the upper BVRF1 extends downward to contact a Tri1 helix (a.a. 298–307) of Tc, then folds back and ascends beyond Tri1 (Fig. 6e, f). Additionally, we also observed some discontinuous densities located at two grooves of triplex Tc: one at the interface between the Tri1 and the Tri2A/B dimer and the other within the two helices from the Tri2B apical region (Fig. 6g). In the unsharpened map of the portal-proximal penton-vertex, these densities could be connected to the N-terminal extension of the upper BVRF1 (Fig. 6h); thus, we assigned the densities to the unmodeled N-terminal residues of the lower BVRF1.

Notably, the interactions in EBV between CATCs and triplexes Ta and Tc are different from those in KSHV.^22^ First, instead of an anchor loop that deeply inserts into a Ta’s hydrophobic cleft between the Tri1 and Tri2A/B dimer, as observed in KSHV pORF32 (BGLF1 homolog), EBV BGLF1 has a helix and an extended loop that contact the Ta surface (Fig. 6b, c). Second, the CATC-Tc interaction in EBV is stronger than that in KSHV, as exemplified by the fact that the contact area between the CATC and Tc in EBV (709 Å^2^) is ~10% larger than that in KSHV (649 Å^2^) (Fig. 6e–h). Those interaction differences appeared to result from the different positioning of the CATC between EBV and KSHV (Supplementary information, Fig. S14). As such, pORF64 (the BVRF1 homolog in KSHV) interacts with one penton MCP at the apex (Supplementary information, Fig. S14a), whereas the head domain of EBV BVRF1 simultaneously contacts two neighboring MCPs at the middle of the MCP tower region (Supplementary information, Fig. S14b). Therefore, the CATC-Ta interactions in KSHV must be more resistant to the “lifting” force exerted by penton expansion than those in EBV, whereas the CATC-Tc interactions in KSHV should be less resistant to the “push” force along the axis of the helix bundle than those in EBV.

## Discussion

The atomic structures of our EBV and other herpesvirus capsids consistently show that their pentons are less strengthened than their hexons, largely due to the variable conformation changes in their penton MCP N-lasso domains, which either adopt an open conformation or are flexible, and thus lose their lashing functionality.^15–18,35^ The N-lasso flexibilities of penton MCPs from different herpesviruses seem inversely proportional to the viral genome sizes. As the viral genome size sequentially increases from HSV-1 (150 kb) to KSHV (165 kb) to EBV (172 kb) to HCMV (235 kb), the N-lasso flexibilities of their penton MCPs decreased in a correlated manner, suggesting that the penton flexibilities also decreased accordingly. Generally, a more stable capsid is necessary for the packaging of a larger viral genome, and therefore, the above observations seem understandable. However, given that the capsids of these herpesviruses are highly pressurized by the packaging of their enormous genomes, what is the underlying reason that herpesviruses still adopt such conformations with a somewhat flexible penton rather than producing a universally stabilized capsid, as in tailed bacteriophages.^26,36,37^

Although the portals of herpesviruses have similar structures to those in bacteriophages, as evidenced by our results reported here (Fig. 3b) as well as other published data,^22,23^ the apparatuses that seal the portal opening in herpesviruses are strikingly different from their bacteriophage counterparts; the portals in bacteriophages are sealed by the assembled tails, whereas the openings of the portals in herpesviruses are covered only by a layer of featureless density called the portal cap (Fig. 1d). It is obvious that the packaged genome within a mature herpesvirus capsid is not as strongly secured as that in a bacteriophage capsid, and therefore, the mechanical elasticity of herpesvirus capsids, mainly conferred by their flexible pentons (Supplementary information, Movie S1), would conceivably reduce the pressure on the portal cap and thus prevent the packaged genome from slipping out the capsid. In addition, the portal itself in a mature herpesvirus capsid also supports the portal cap. In bacteriophages, it has been shown that the portal in the tailed infectious virus has an open conformation,^31^ whereas our result with EBV shows that the in situ portal adopts a much more compacted conformation than that of the in vitro-assembled portal (Supplementary information, Fig. S12 and Movie S1). Consequently, the terminal DNA of the packaged genome in the mature EBV capsid is firmly held by the significantly narrowed portal channel valve, which would further alleviate the pressure placed on the portal cap. It is noteworthy that compared to that of the mature intact HSV-1 and KSHV virions, each of which is encircled by only one layer of DNA, the crown region of the tegumented EBV capsid portal is tightly associated with three layers of DNA densities (Supplementary information, Fig. S10b and Movie S1), which likely function to “brake” the DNA slipping with increased efficiency. In the natural process of herpesvirus infection, the intact virions enter the host cell through membrane fusion to produce the de-enveloped, tegumented nucleocapsids, which are then trafficking to the nucleopore for genome ejection. Without the protection of the viral envelope and outer tegument layer, the viral portal cap is less resistant to the capsid inner pressure and thus needs further support from the portal for genome retention.

It has been demonstrated that the genome release of a herpesvirus capsid is pressure dependent.^38^ Therefore, it is necessary to maintain an appropriately high inner pressure for herpesvirus capsids. Compared with the occupancy (37.9%) of the penton-vertex registers in KSHV,^22^ the CATC occupancy (20.3%) in EBV is significantly lower, even though EBV contains a larger DNA genome. In addition, some of the penton vertices in both the KSHV (7.3%) and EBV (37.9%) mature nucleocapsids lack CATCs and thus remain vulnerable.^39^ According to the wooden barrel theory, the CATCs in both KSHV and EBV should have little or no effects on the overall stability of viral capsids. Instead, through binding to the penton-vertex registers in a stoichiometric manner, the CATCs could accordingly reduce the overall capsid flexibility and thus precisely adjust the capsid inner pressure to achieve a balance between genome retention (the lower pressure is, the better) and efficient ejection (the higher pressure is, the better). Such a pressure-regulating function of CATCs is also at play in HSV-1 and HSV-2: the full occupancy of CATCs^17,35^ at penton vertices not only enhances capsid stability that possibly benefit the long-distance cellular transport in neuronal cells but also increases inner capsid pressure to efficiently release the genome, which is obviously smaller than that of EBV or KSHV.^38^

## Materials and methods

### EBV virion preparation

We modified the culture method of EBV from the previously described procedures.^40^ Briefly, the EBV-transformed B95-8 marmoset cell line^25^ was cultured in RPMI 1640 medium supplemented with 10% heat-inactivated FBS (heated at 56 °C for 0.5 h), 100 U/mL penicillin and 100 μg/mL streptomycin and was incubated in a humidified incubator with 5% CO_2_. During the initial 4 days, fresh medium at one-half of the cell-culture volume was added into the flask every day. The concentration of the growing cells was monitored by a cell counting instrument (CountStar^®^). When it reached a density of ~10^6^ cells/mL, 20 ng/mL tetradecanoyl phorbol acetate (TPA) was added to induce the EBV lytic cycle.^41^ Two hours later, the cells were washed three times with fresh RPMI 1640 to remove TPA and then resuspended in RPMI 1640 medium containing 5% heat-inactivated FBS, 100 units/mL penicillin and 100 μg/mL streptomycin in 3.5% CO_2_. To ensure that the medium did not become too acidic, the TPA-induced cells were then cultured with gradually decreased CO_2_ concentrations (0.5% per day). At 4 days post TPA induction, when a majority of cells were lysed, the supernatant was collected by centrifuging the culture medium at 10,000 × *g* for 10 min to remove the cell debris. The EBV particles were then pelleted by centrifuging the supernatant at 80,000 × *g* for 1 h. After resuspended in phosphate-buffered saline (PBS, pH 7.4), the pellet was further purified by centrifugation through a 15%–50% (w/v) continuous sucrose gradient at 100,000 × *g* for 1 h. The light-scattering band containing EBV particles was collected and diluted with PBS to a volume of 13 mL. Finally, the EBV particles were pelleted by centrifugation at 80,000 × *g* for 1 h to remove the sucrose and resuspended in 10 μL of PBS.

### CryoEM sample preparation and data acquisition

CryoEM samples were prepared immediately after mixing the purified intact EBV virions with Tween-100 at a final concentration of 0.8% to break down the viral envelope. A 2.5 μL aliquot of the viral sample was applied to a glow-discharged 200-mesh Quantifoil grid (R2/1), blotted with filter paper for 12.0 s and frozen by plunging into liquid ethane using a FEI Vitrobot IX. CryoEM micrographs were collected on a 300 kV Titan Krios microscope (FEI) equipped with a Gatan Imaging filter (GIF) and a K2 summit direct detection camera. The microscope was operated at 300 kV with a nominal magnification of 105,000×, yielding a calibrated pixel size of 1.31 Å on specimen. A total of 16,621 movies were collected using the software package SerialEM at a dose rate of 8 electrons/Å^2^ s for 6 s.

### CryoEM image processing and icosahedral reconstruction

For each movie stack, the 24 frames were aligned by beam-induced motion correction with the program MotionCor2.^42^ The defocus values and astigmatism parameters for each micrograph were determined by Gctf.^43^ A total of 36,121 well-separated DNA-containing particles were picked manually by using Manual Picking in Relion 3.0.^44^ To spare computational resources, the boxed particle images (1280 × 1280) were binned 8 times before being subjected to 2D and 3D classifications. 3D classifications were performed using a “Gaussian ball” as the reference. A total of 32,721 particles from a good 3D class were extracted without binning for 3D refinement. A cryoEM structure of the EBV capsid at a 4.6 Å resolution was obtained by conventional icosahedral reconstruction in Relion 3.0. To correct the Ewald-sphere curvature for such a massive particle (1280 Å), we added the argument –Ewald in the relion_reconstruction program in Relion 3.0 for both half maps. The final resolution of the icosahedral reconstruction was improved to 4.1 Å, as calculated from the two Ewald-corrected half maps. The global and local resolutions for all reconstructions were determined by Golden-standard Fourier shell correlation using the 0.143 threshold^45^ and ResMap,^46^ respectively.

### Classification and refinement of the portal-vertex and portal sub-particles and asymmetric reconstruction of the capsid

We determined the nonicosahedral symmetric structures of the portal vertex, portal and capsid of EBV by following a strategy similar to that used for HSV-1,^23^ as illustrated in Supplementary information, Fig. S2. Briefly, 12 vertex sub-particles of each capsid were extracted using the icosahedral orientation and center parameters of the capsid as guidance.^47^ The vertex sub-particles were then subjected to 3D classification with 5-fold symmetry without alignment of rotations by using the –skip_rotate argument in Relion 3.0. Two of the six classes, which in total accounted for 7.7% of the sub-particle dataset and showed prominent portal features, were significantly different from the other four classes, each of which displayed a penton at the center. After removing the redundant sub-particles, we performed 3D refinement with 5-fold symmetry imposed and obtained a reconstruction at a resolution of 4.2 Å from a total of 28,639 portal-vertex sub-particles.

To determine the dodecameric portal structure, we further extracted the sub-particles including only the dodecameric portal part from the portal-vertex sub-particles and expanded the 5-fold symmetry of the dodecamer sub-particles using relion_particle_symmetry_expand. Then, the 5-fold-expanded sub-particles were subjected to 3D classification with C12 symmetry imposed without rotation alignment. While five of the six generated classes showed a prominent portal structure containing 15.9%, 19.1%, 20.3%, 19.5% and 20.6% of the symmetry-expanded sub-particle dataset, the other one, accounting for 4.6% of the dataset, failed to present any structural features, possibly due to unsuccessful searching of the centers of those expanded sub-particles. Of the four portal classes, we selected the one with the ratio closest to 20% of the whole dataset. After removing the redundant particles, we finally obtained a total of 22,782 portal sub-particles. By imposing C12 symmetry, we refined the dodecameric reconstruction of the portal to a 4.8 Å resolution. To determine the asymmetric structures of the portal vertex and the capsid, we applied the orientations of the above 22,782 portal sub-particles to the corresponding portal-vertex sub-particles and capsid particles and then performed 3D local refinement without imposing any symmetry. By doing so, we finally obtained the portal-vertex and the capsid reconstructions at resolutions of 5.5 Å and 7.4 Å, respectively.

### Classifications and refinements of the CATC-absent penton-vertex and CATC-binding portal-proximal penton-vertex in EBV

The portal-vertex classification described above generated two classes of portal vertexes and four penton-vertex classes that are together responsible for 92.3% of the vertex sub-particles (Supplementary information, Fig. S2). We initially performed 3D refinement of the penton-vertex sub-particles with 5-fold symmetry imposed to determine more accurate center parameters for each sub-particle, and we obtained a reconstruction of the penton vertex at a resolution of 3.5 Å. Since the CATC might occupy any of the five registers at the penton vertex with the capsid proteins in a 5-fold symmetry arrangement, we expanded the 5-fold symmetry of the refined sub-particles and performed a round of focused classification (without searching any orientations by using –skip_align) of the symmetry-expanded sub-particles with a mask containing only one CATC. A –tau value of 20 was used for the masked classification. Among the resulted 4 classes, one class (20.3% of the symmetry-expanded dataset) had CATC densities as strong as those of the surrounding capsid proteins, whereas the remaining three classes clearly lacked CATC densities. We thus treated those three classes as CATC-absent classes. To determine the structure of the CATC-absent penton vertex, we removed the duplicated sub-particles to ensure that only one of the five symmetry-expanded sub-particles was retained and removed the sub-particles belonging to the CATC-binding class. Finally, 137,356 particles were used for the final reconstruction of the CATC-absent penton vertex with 5-fold symmetry imposed at a resolution of 3.8 Å.

The C1 capsid reconstruction revealed CATC densities binding only a specific one of the five registers at the portal-proximal penton vertex. To determine the high-resolution CATC structure at the penton vertex, we extracted 113,910 portal-proximal penton-vertex sub-particles and carried out classification with a mask encompassing only the bound CATC. Finally, 93,334 (83.3%) portal-proximal penton-vertex sub-particles containing a copy of CATC were used for the final reconstruction of the CATC-absent penton vertex without symmetry, and the resolution was determined to be 4.3 Å.

### Model building

One asymmetric unit of the icosahedral capsid of EBV contains 46 unique copies of the four capsid proteins: 16 MCPs and 16 SCPs from C-hexon, P-hexon, E-hexon and penton, 5 Tri1s and 10 Tri2s from 5 subtypes of triplexes Ta to Te. To guarantee good geometry and validity of our models, we initially used our high-resolution C5 map of the penton vertex at a resolution of 3.5 Å to ab initio build models in COOT^48^ of the components in the penton-vertex region (including hexon MCPs and SCPs P1, P2, P5 and P6; penton MCPs and SCPs; and triplexes Ta and Tc). Then, the structures of one copy of MCP, SCP and triplex were fitted into every quasiequivalent position in the asymmetric unit of our icosahedral reconstruction of the capsid and manually adjusted in COOT to improve their correspondence to our density map. After refined models of all unique conformers in an asymmetric unit were obtained, iterations of real space refinement within PHENIX (phenix.real_space_refine)^49^ were applied to the combined models of all conformers to optimize the atomic models. To build an atomic model of the portal-vertex region, we fitted the models of peripenton capsid proteins, including SCP-bound hexon MCPs (P1, P2, P5 and P6) and triplexes Ta and Tc, into our C5 density map of the portal vertex with Chimera.^50^ The models of capsid proteins were then manually adjusted in COOT. In addition, we traced and modeled the CATC structures ab initio with the help of secondary structure predictions from Phyre2.^51^ Finally, the atomic models of periportal capsid proteins and the bound CATCs were combined and refined together in PHENIX. To construct a homology model of the BVRF1 head domain, we docked the crystal structure of the HSV-1 pUL25 head domain (PDB: 2F5U)^33^ into one of the two globular densities with stronger intensity of the C1 reconstruction of the portal-proximal penton vertex, manually adjusted the placement in COOT and then refined it in PHENIX. To model the portal protein BBRF1, we fitted the BBRF1 monomer structure (PDB: 6RVS)^28^ derived from the in vitro-assembled EBV portal into our C12 portal density map in Chimera, manually adjusted the fit in COOT and then refined it in PHENIX.

## Supplementary information

Supplementary information, Movie S1

Supplementary information, Table S1

Supplementary information, Table S2

Supplementary information, Fig. S1

Supplementary information, Fig. S2

Supplementary information, Fig. S3

Supplementary information, Fig. S4

Supplementary information, Fig. S5

Supplementary information, Fig. S6

Supplementary information, Fig. S7

Supplementary information, Fig. S8

Supplementary information, Fig. S9

Supplementary information, Fig. S10

Supplementary information, Fig. S11

Supplementary information, Fig. S12

Supplementary information, Fig. S13

Supplementary information, Fig. S14

## Data Availability

All density maps have been deposited in the Electron Microscopy Bank under accession codes EMD-30162 (icosahedral capsid), EMD-30157 (C5 portal vertex), EMD-30155 (C12 portal), EMD-30146 (C1 capsid), EMD-30156 (C1 portal vertex), EMD-30152 (C5 penton vertex), EMD-30158 (C1 CATC-binding portal-proximal penton vertex) and EMD-30159 (C5 CATC-absent penton vertex), respectively. The atomic coordinates have been deposited in the Protein Data Bank under accession code 7BSI (the icosahedral capsid), 7BQT (C12 portal), 7BQX (C5 portal vertex), 7BR7 (C1 CATC-binding portal-proximal penton vertex) and 7BR8 (C5 CATC-absent penton vertex), respectively.
