# Supplementary material for: CryoEM structure of the tegumented capsid of Epstein-Barr virus
Source: Cell Res. 2020 Jul 3;30(10):873–84. doi: 10.1038/s41422-020-0363-0 (PMC7608217; doi:10.1038/s41422-020-0363-0)
Supplement: Supplementary file 2 — Supplementary information, Table S1 [file 41422_2020_363_MOESM2_ESM.pdf]

Supplementary information, Table. S1| Cryo-EM data collection, image processing, and refinement statistics

	Icosahedral capsid (EMD:; PDB:)	C5 penton vertex (EMD:;)	C5 portal vertex (EMD:; PDB:)	C12 portal (EMD:; PDB:)	C5 CATC-absent penton vertex (EMD:; PDB:)	C1 CTAC-binding proximal -portal penton vertex (EMD:; PDB:)	C1 portal vertex (EMD:)	C1 capsid (EMD:)
**Data collection and processing**								
Voltage (kV)	300	300	300	300	300	300	300	300
Electron exposure (e–/Å^2)	30	30	30	56	56	56	56	40
Defocus range (μm)	-0.5 to -2.0	-0.5 to -2.0	-0.5 to -2.0	-0.5 to -2.0	-0.5 to -2.0	-0.5 to -2.0	-0.5 to -2.0	-0.5 to -2.0
Pixel size (Å)	1.31	1.31	1.31	1.31	1.31	1.31	1.31	1.31
Symmetry imposed	I2	C5	C5	C12	C5	C1	C1	C1
Initial particle images (no.)	36,121	392,652	30,234	29,068	362,417	362,417	28,639	32,721
Final particle images (no.)	32,721	362,417	28,639	22,782	137,356	93,334	22,782	22,782
Map resolution (Å)	4.1	3.5	4.2	4.8	3.8	4.3	5.5	7.4
FSC threshold	0.143	0.143	0.143	0.143	0.143	0.143	0.143	0.143
Map resolution range (Å)	4.0-8.0	3.5-6.0	3.5-6.0	4.0-7.0	3.5-6.0	4.0-7.0	4.5-8.0	6.0-9.0
**Refinement**								
Initial model used (PDB code)	6B43	6B43	6PPB	6RVS	6B43	6PPB	n/a	n/a
Model resolution (Å)	4.1	n/a	4.4	4.8		4.1	n/a	n/a
FSC threshold	0.5		0.5	0.5	0.5	0.5		
Model resolution range (Å)	4.0-8.0		3.5-6.0	4.0-7.0	3.5-6.0	4.0-7.0		
Map sharpening *B* factor (Å^2)	-195	-175	-180	-357	-198	-182	-179	-310
Model composition		n/a					n/a	n/a
Non-hydrogen atoms	214,177		61,830	39,180	68,422	73,131		
Protein residues	27180		7,843	4860	8,678	9,266		
Ligands	0		0	0	0	0		
B factors (Å^2)								
Protein	106		88	157	47	120		
Ligand					0			
R.m.s. deviations								
Bond lengths (Å)	0.006		0.007	0.006	0.006	0.006		
Bond angles (°)	0.947		1.024	1.061	0.918	0.914		
Validation								
MolProbity score	1.85		1.90	1.97	1.77	1.86		
Clashscore	7.80		8.19	11.04	6.58	7.63		
Poor rotamers (%)	0.51		0.89	0.66	0.56	0.58		
Ramachandran plot								
Favored (%)	94.27		92.84	94.03	94.51	93.35		
Allowed (%)	5.72		7.16	5.97	5.48	6.65		
Disallowed (%)	0.01		0.00	0.00	0.01	0.00		
