# Supplementary material for: CryoEM structure of the tegumented capsid of Epstein-Barr virus
Source: Cell Res. 2020 Jul 3;30(10):873–84. doi: 10.1038/s41422-020-0363-0 (PMC7608217; doi:10.1038/s41422-020-0363-0)
Supplement: Supplementary file 3 — Supplementary information, Table S2 [file 41422_2020_363_MOESM3_ESM.pdf]

Supplementary information, Table. S2| Information of the EBV proteins involved in the formation of the capsid

Protein name in EBV	Common name in herpesviruses	length
BcLF1	Major capsid protein (MCP)	1382
BFRF3	Small capsid protein (SCP)	177
BORF1	Triplex monomer (Tri1)	364
BDLF1	Triplex dimer (Tri2)	301
BBRF1	Portal protein	614
BVRF1	CATC	571
BPLF1	CATC	3149
BGLF1	CATC	508
