# Supplementary material for: CryoEM structure of the tegumented capsid of Epstein-Barr virus
Source: Cell Res. 2020 Jul 3;30(10):873–84. doi: 10.1038/s41422-020-0363-0 (PMC7608217; doi:10.1038/s41422-020-0363-0)
Supplement: Supplementary file 4 — Supplementary information, Fig. S1 [file 41422_2020_363_MOESM4_ESM.pdf]

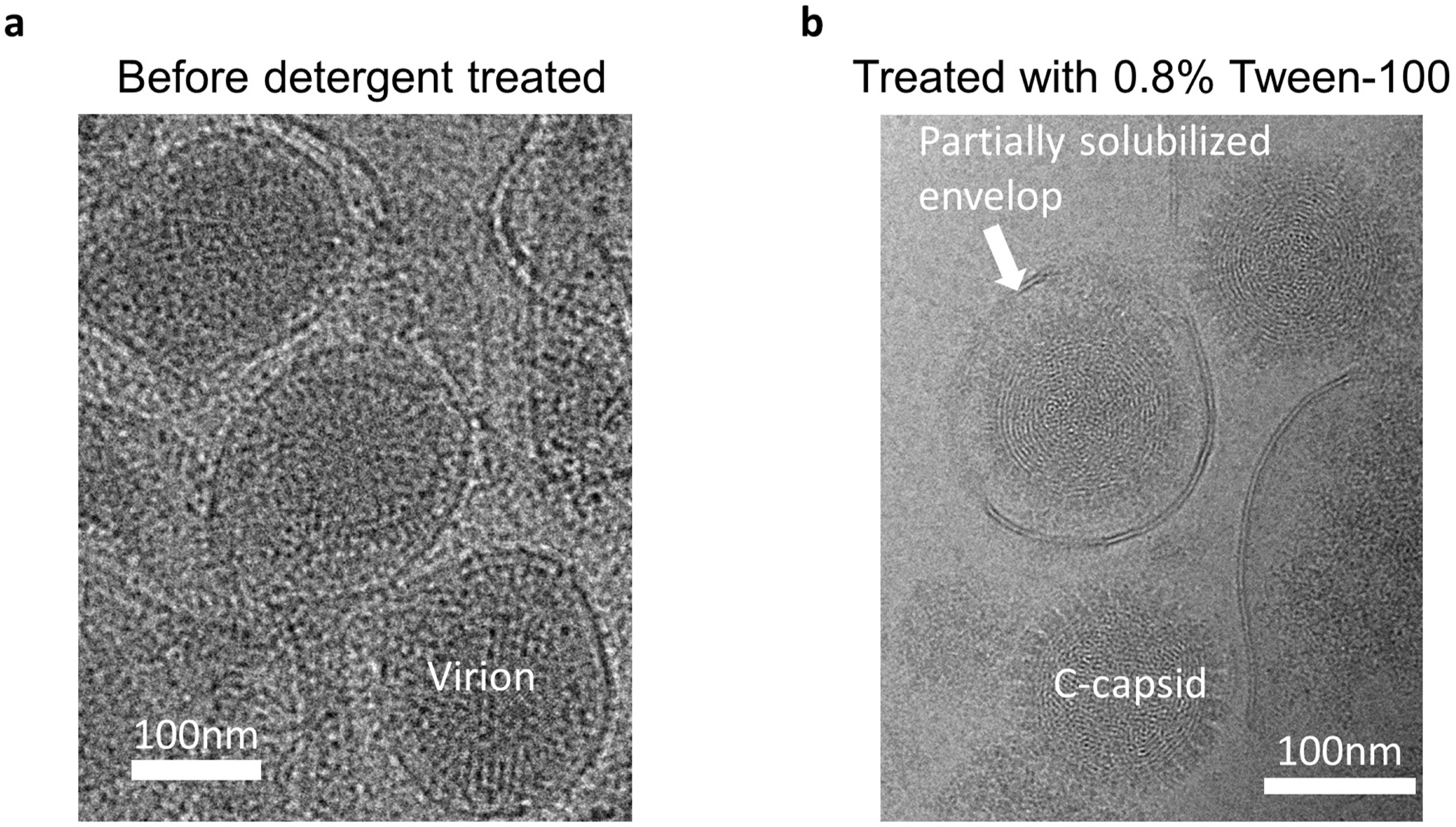

Supplementary information, Fig. S1| CryoEM micrographs of EBV virion.

a A representative image recorded with a 200 kV Talos Arctica microscope, showing intact EBV virions before the detergent treatment.

b A representative image recorded with 300 kV Titan Krios microscope, showing that the viral envelopes were removed or partially solubilized by detergent treatment.
