# Supplementary material for: CryoEM structure of the tegumented capsid of Epstein-Barr virus
Source: Cell Res. 2020 Jul 3;30(10):873–84. doi: 10.1038/s41422-020-0363-0 (PMC7608217; doi:10.1038/s41422-020-0363-0)
Supplement: Supplementary file 5 — Supplementary information, Fig. S2 [file 41422_2020_363_MOESM5_ESM.pdf]

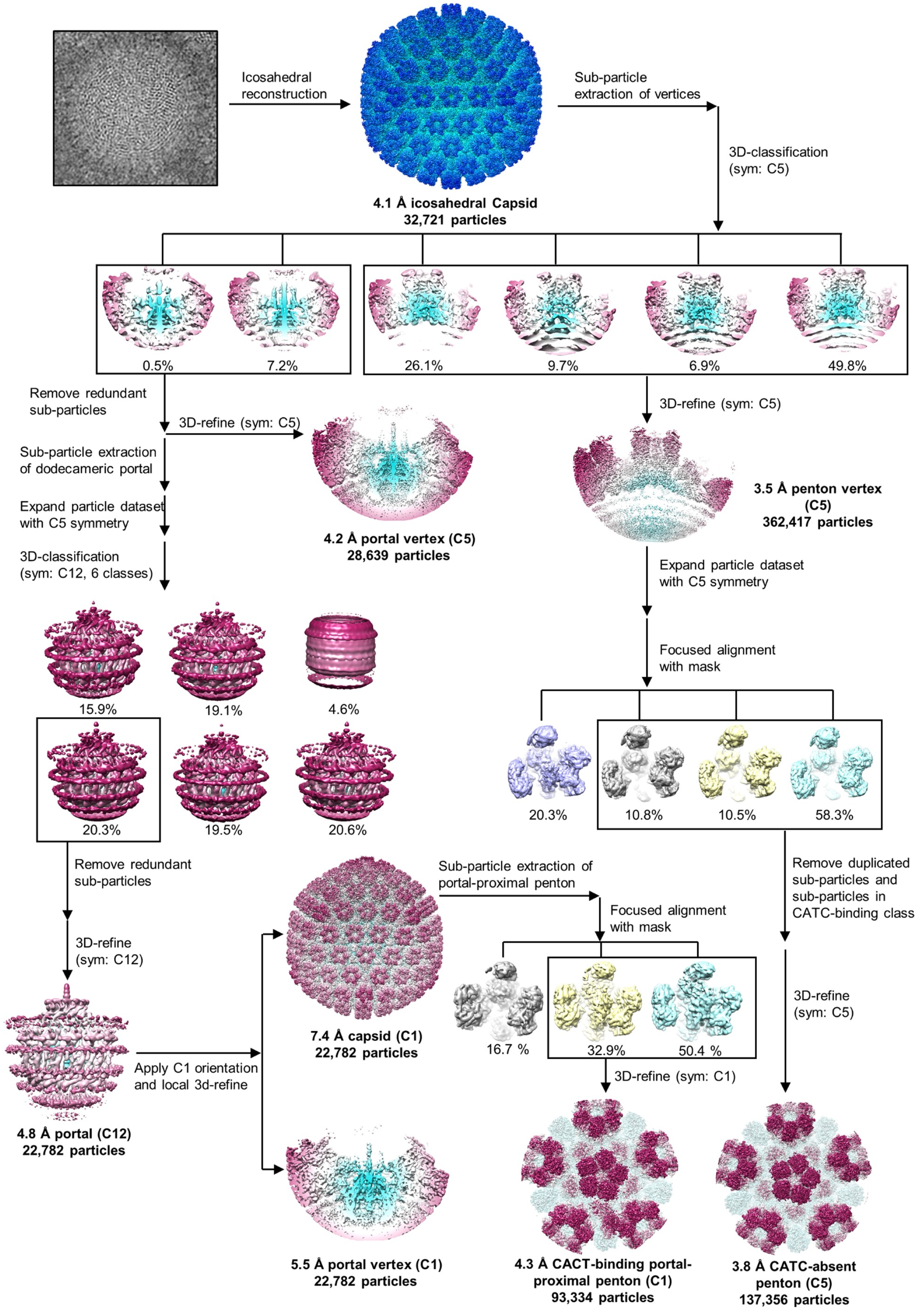

Supplementary information, Fig. S2| Icosahedral reconstruction of EBV capsid and sequential 3D classification and refinement of sub-particles.
