# Supplementary material for: CryoEM structure of the tegumented capsid of Epstein-Barr virus
Source: Cell Res. 2020 Jul 3;30(10):873–84. doi: 10.1038/s41422-020-0363-0 (PMC7608217; doi:10.1038/s41422-020-0363-0)
Supplement: Supplementary file 6 — Supplementary information, Fig. S3 [file 41422_2020_363_MOESM6_ESM.pdf]

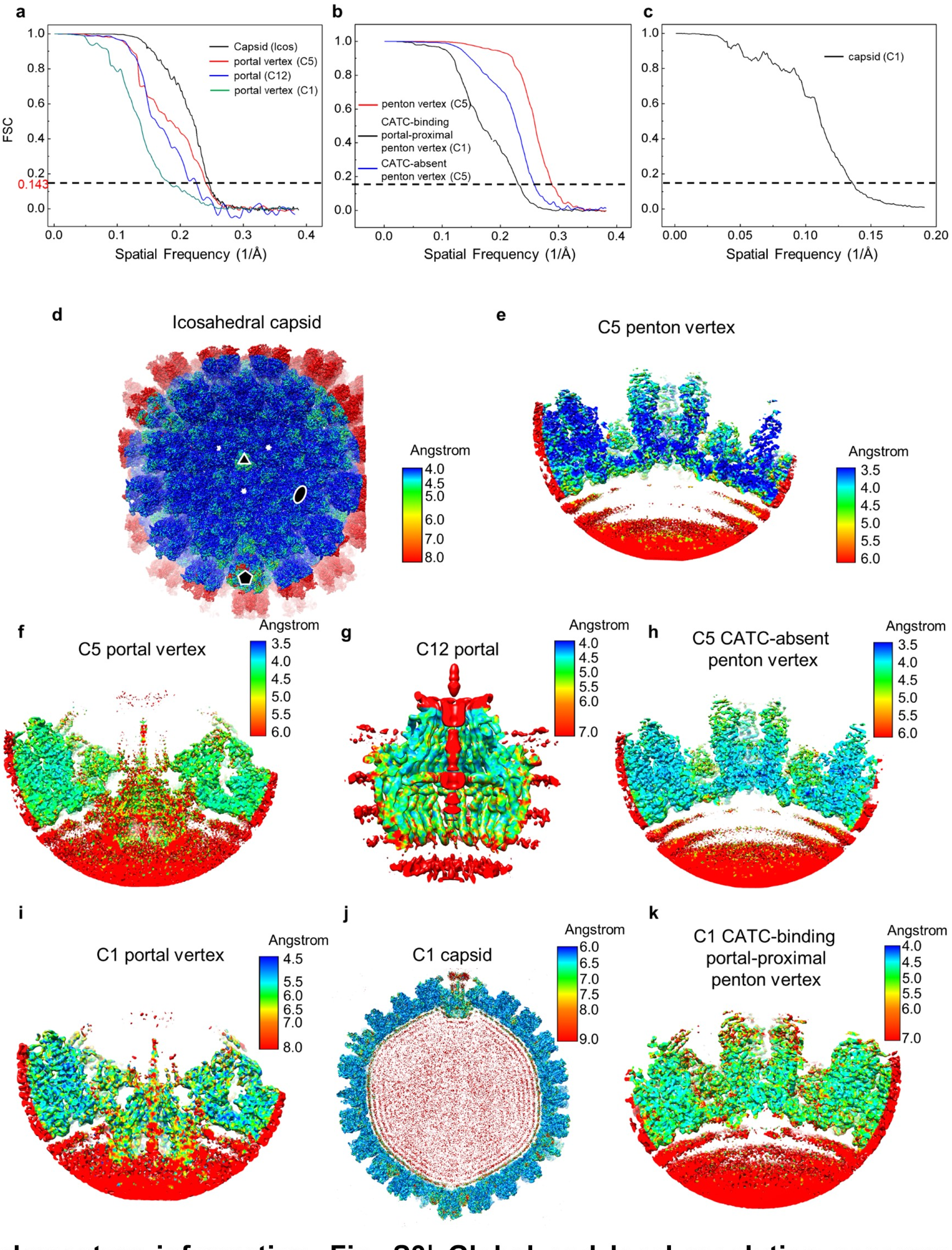

Supplementary information, Fig. S3| Global and local resolution assessments of reconstructions.

a-c Gold-standard FSC curves of cryoEM reconstructions, including the icosahedral capsid (4.1 Å), C5 portal vertex (4.2 Å), C5 penton vertex (3.5 Å), C12 portal (4.8 Å), C1 portal vertex (5.5 Å), C5 CATC-absent penton vertex (3.8 Å), C1 CATC-binding portal-proximal penton vertex (4.3 Å) and C1 capsid (7.4 Å).

d-k Local resolution distributions of density maps estimated by ResMap. In (d), a sub-volume at a dimension of 600 x 600 x 600 pixels of the icosahedral capsid map was subjected to local resolution assessment. The 5-fold, 3-fold and 2-fold axes are denoted by a pentagon, triangle and oval, respectively.
