# Supplementary material for: CryoEM structure of the tegumented capsid of Epstein-Barr virus
Source: Cell Res. 2020 Jul 3;30(10):873–84. doi: 10.1038/s41422-020-0363-0 (PMC7608217; doi:10.1038/s41422-020-0363-0)
Supplement: Supplementary file 8 — Supplementary information, Fig. S5 [file 41422_2020_363_MOESM8_ESM.pdf]

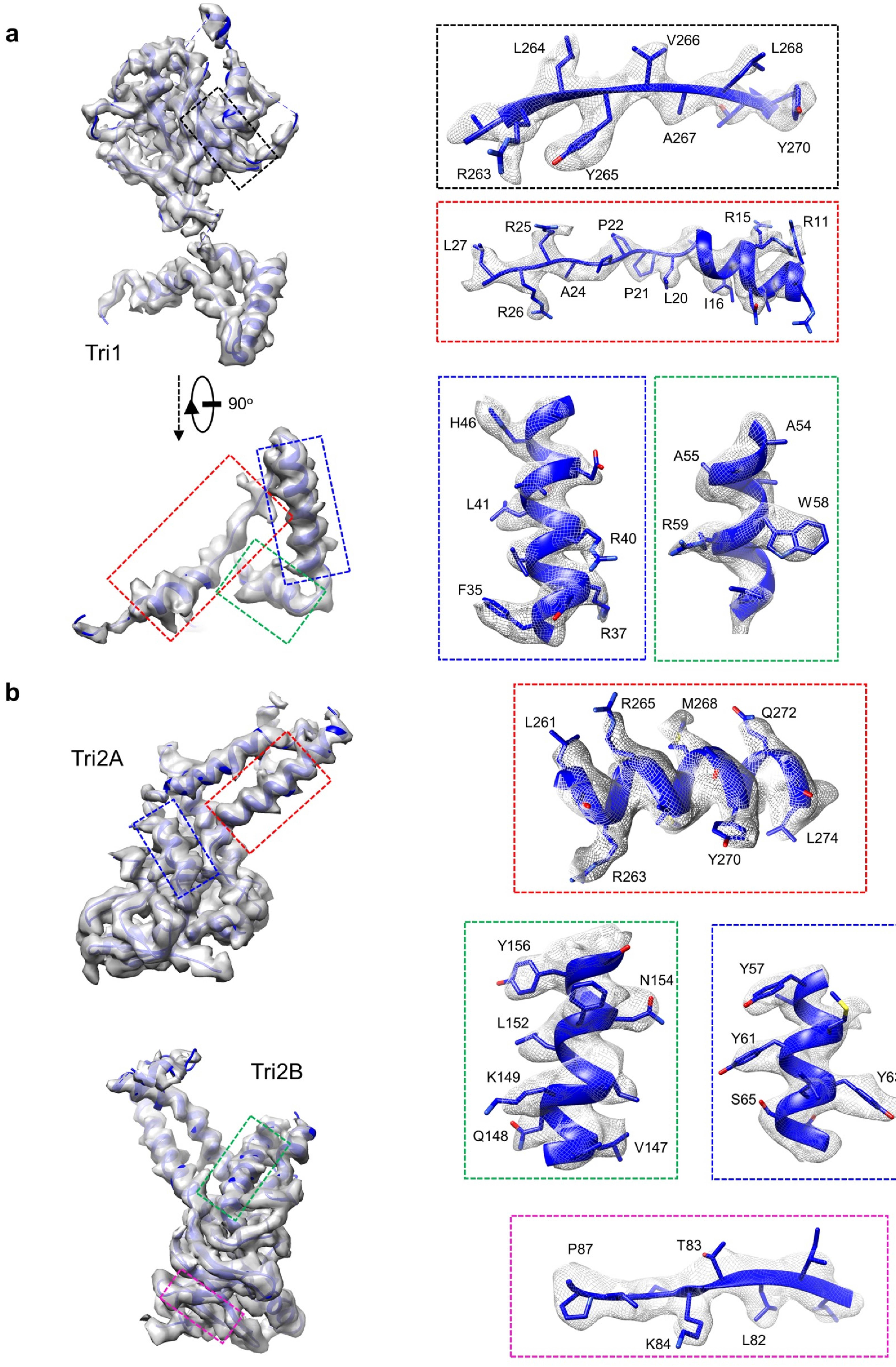

Supplementary information, Fig. S5| Density maps from the 3.5 Å resolution map of the penton vertex and atomic models of triplex heterotrimer—Tri1 (a), Tri2A and Tri2B (b)

The densities were derived from the 3.5 Å resolution reconstruction of the penton vertex. The insets are zoomed-in views of the boxed regions to illustrate the side-chain features in the density maps.
