# Supplementary material for: CryoEM structure of the tegumented capsid of Epstein-Barr virus
Source: Cell Res. 2020 Jul 3;30(10):873–84. doi: 10.1038/s41422-020-0363-0 (PMC7608217; doi:10.1038/s41422-020-0363-0)
Supplement: Supplementary file 9 — Supplementary information, Fig. S6 [file 41422_2020_363_MOESM9_ESM.pdf]

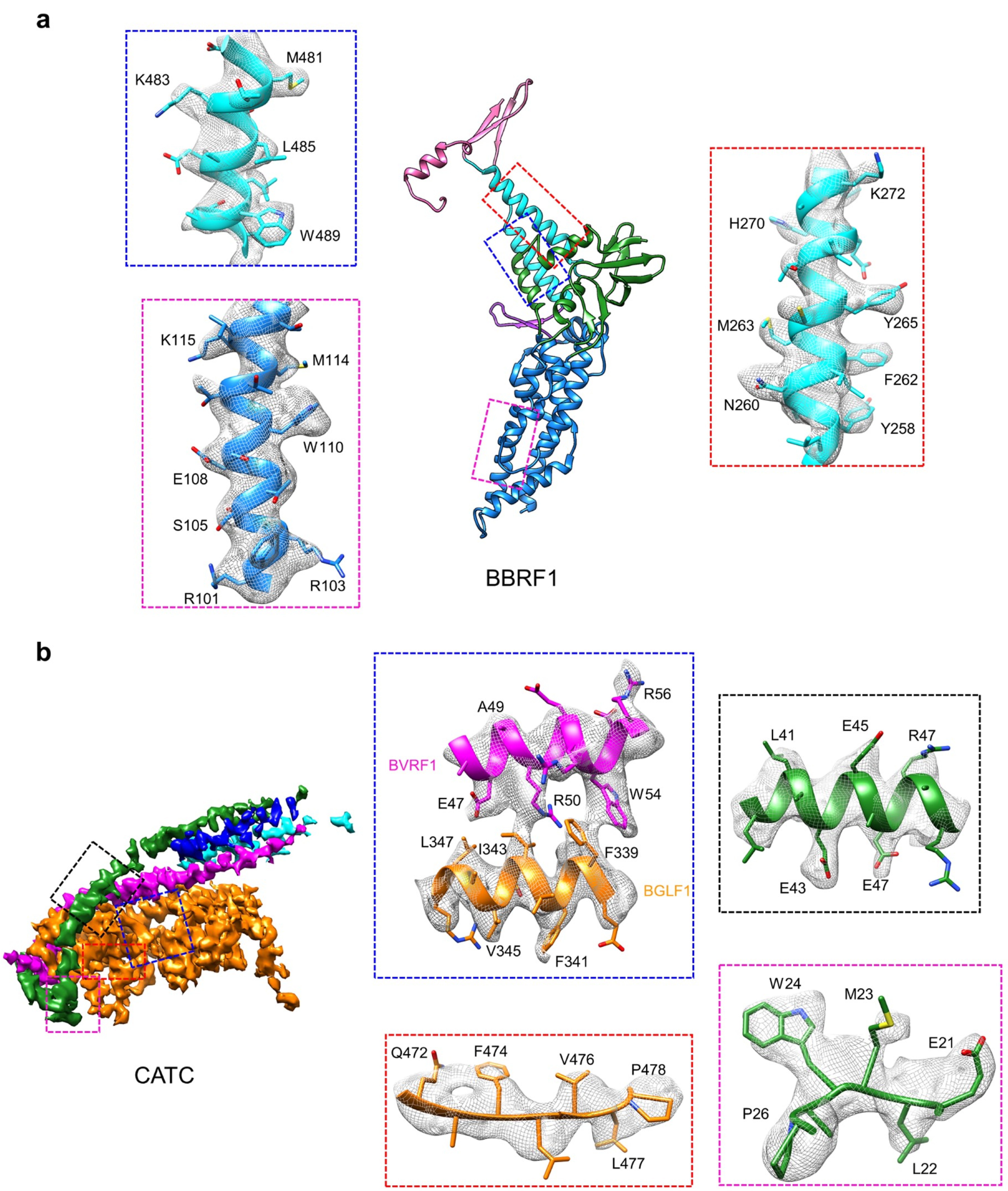

Supplementary information, Fig. S6| Density maps and atomic models of BBRF1 and CATC.

a BBRF1 density map and atomic model.

b Density maps and atomic models of the CATC in the portal-proximal penton vertex. Insets are zoomed-in views of the boxed regions to show the residue features in the density maps.
