# Supplementary material for: CryoEM structure of the tegumented capsid of Epstein-Barr virus
Source: Cell Res. 2020 Jul 3;30(10):873–84. doi: 10.1038/s41422-020-0363-0 (PMC7608217; doi:10.1038/s41422-020-0363-0)
Supplement: Supplementary file 10 — Supplementary information, Fig. S7 [file 41422_2020_363_MOESM10_ESM.pdf]

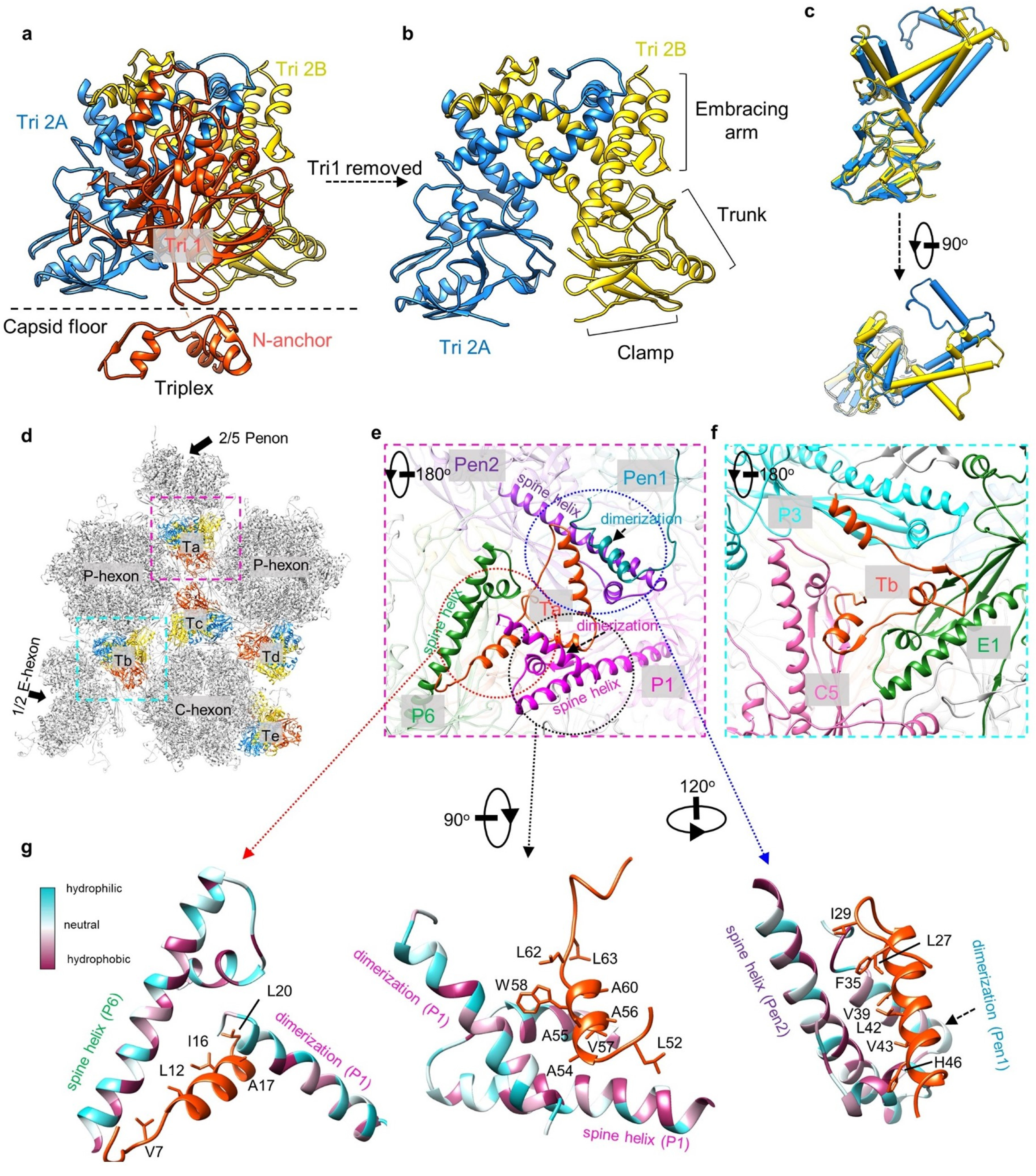


**Supplementary information, Fig. S7| Triplex structure and interactions with MCP.**

**a** Triplex structure.

**b** Models of Tri2A (blue) and Tri2B (yellow), showing that the two conformers embrace each other.

**c** Superposition between Tri2A and Tri2B, showing that the two conformers have nearly identical clamp and trunk domains but significantly different embracing arms.

**d** Overview of triplexes and MCPs.

**e-f** Zoomed-in views in (b) from capsid interior, showing the N-anchoring of triplex Ta (e) and Tb (f), respectively, to the capsid floor.

**g** Hydrophobic interactions between N-anchor of Ta Tri1 and capsid proteins. The hydrophobic residues of helices in N-anchor are highlighted by showing side chains. The helices in capsid proteins are colored according to the hydrophobicity of their residues.
