# Supplementary material for: CryoEM structure of the tegumented capsid of Epstein-Barr virus
Source: Cell Res. 2020 Jul 3;30(10):873–84. doi: 10.1038/s41422-020-0363-0 (PMC7608217; doi:10.1038/s41422-020-0363-0)
Supplement: Supplementary file 11 — Supplementary information, Fig. S8 [file 41422_2020_363_MOESM11_ESM.pdf]

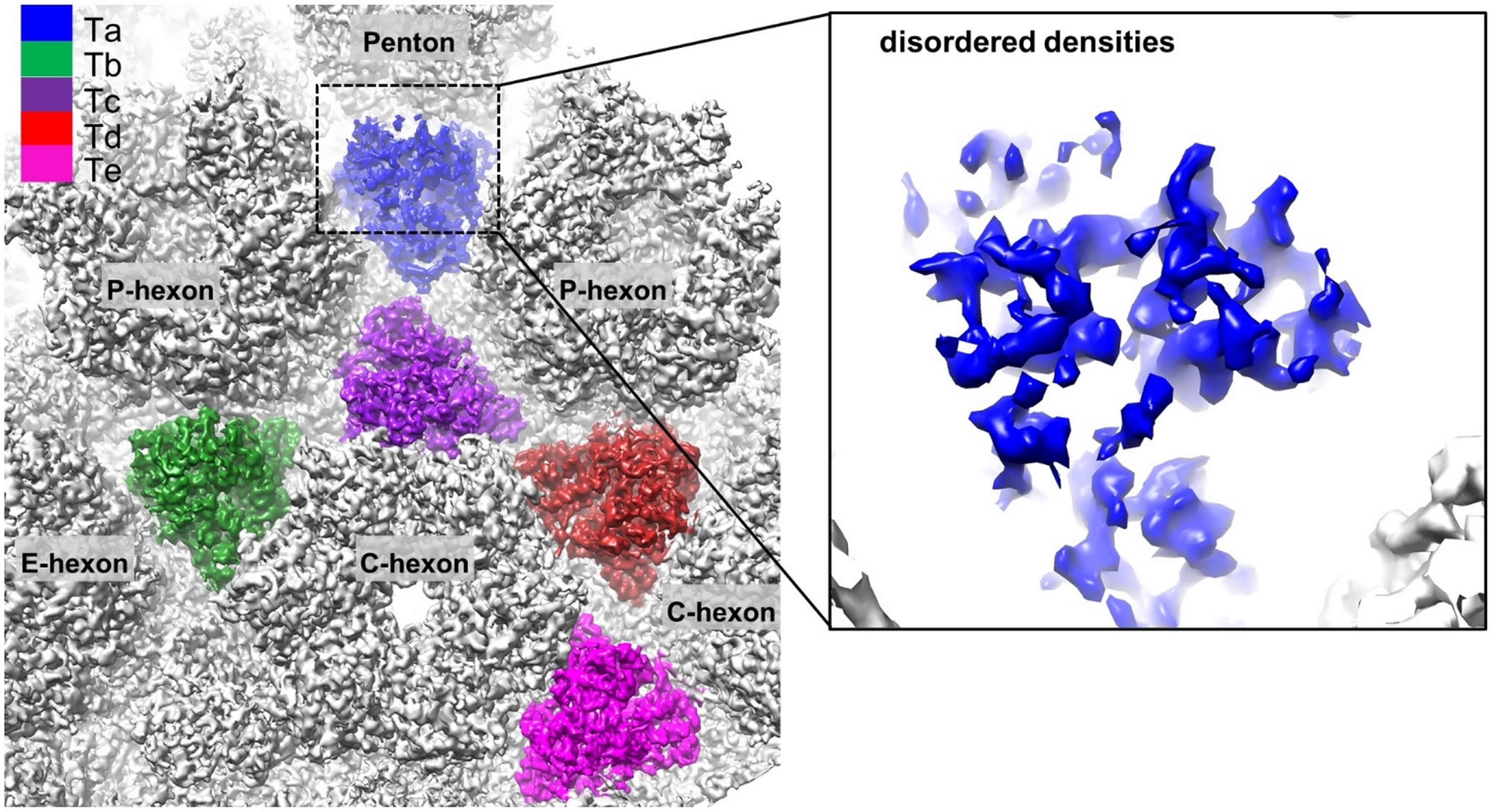

Supplementary information, Fig. S8| Density map of a selected region from the icosahedral capsid reconstruction. The MCPs and SCPs are in gray. The triplexes are colored according to the key. The inset is the zoomed-in view of the boxed regions to indicate the disordered densities of Ta.
