# Supplementary material for: CryoEM structure of the tegumented capsid of Epstein-Barr virus
Source: Cell Res. 2020 Jul 3;30(10):873–84. doi: 10.1038/s41422-020-0363-0 (PMC7608217; doi:10.1038/s41422-020-0363-0)
Supplement: Supplementary file 12 — Supplementary information, Fig. S9 [file 41422_2020_363_MOESM12_ESM.pdf]

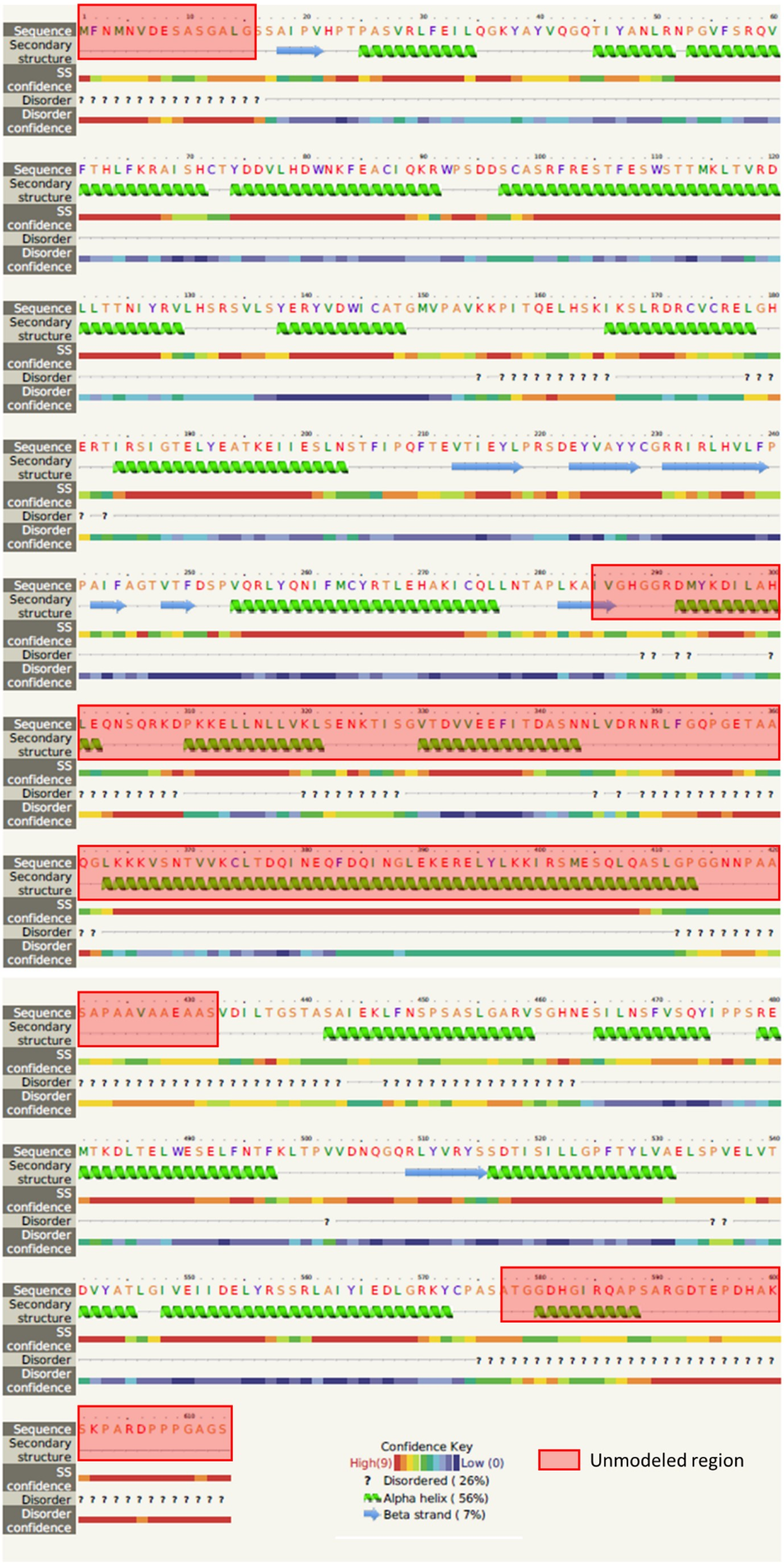


**Supplementary information, Fig. S9| Secondary structure and disorder prediction of BBRF1 protein.**

The prediction was performed using the *Phyre2*. The red boxes indicated the regions that were not modeled in our BBRF1 structure.
