# Supplementary material for: CryoEM structure of the tegumented capsid of Epstein-Barr virus
Source: Cell Res. 2020 Jul 3;30(10):873–84. doi: 10.1038/s41422-020-0363-0 (PMC7608217; doi:10.1038/s41422-020-0363-0)
Supplement: Supplementary file 13 — Supplementary information, Fig. S10 [file 41422_2020_363_MOESM13_ESM.pdf]

a

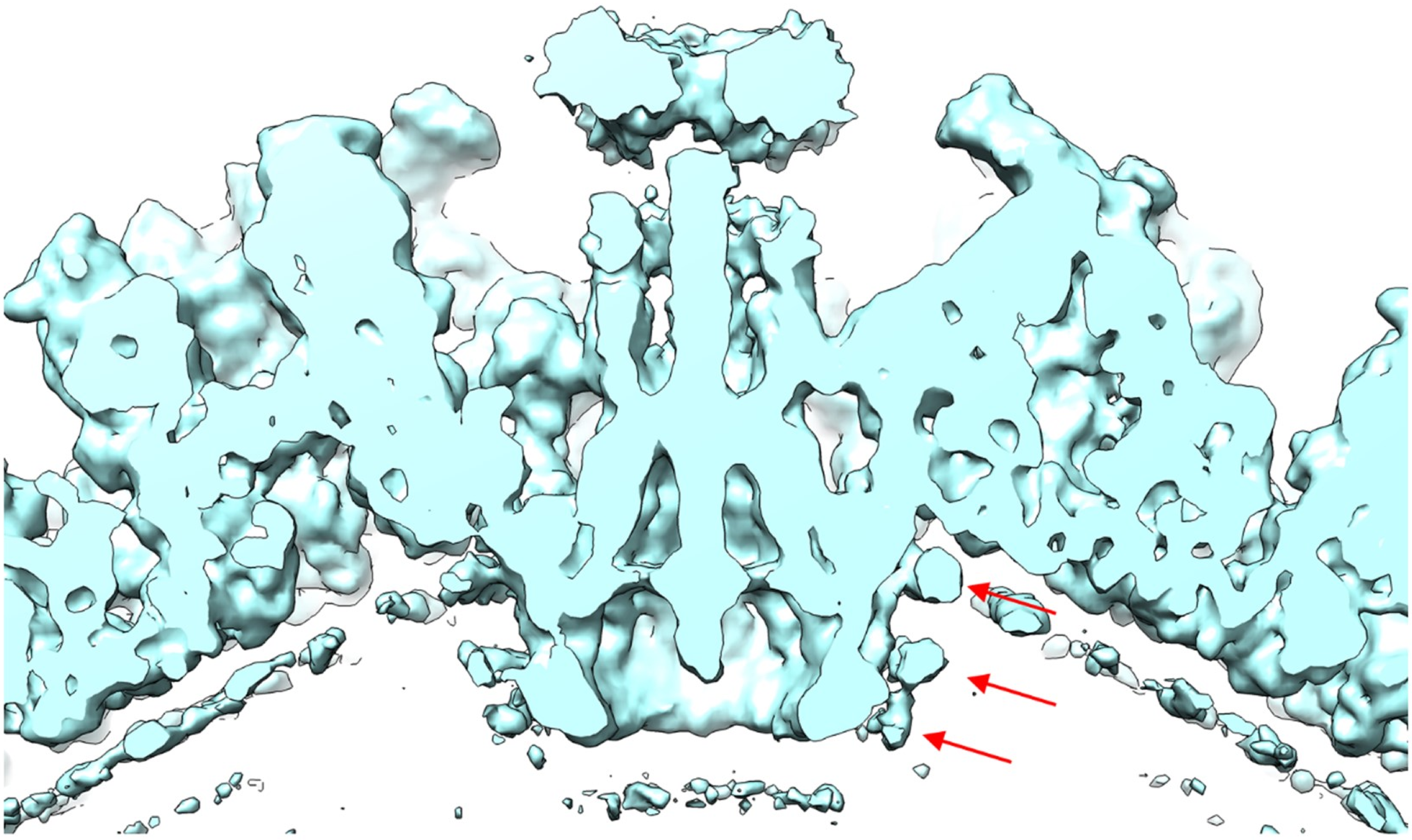

b

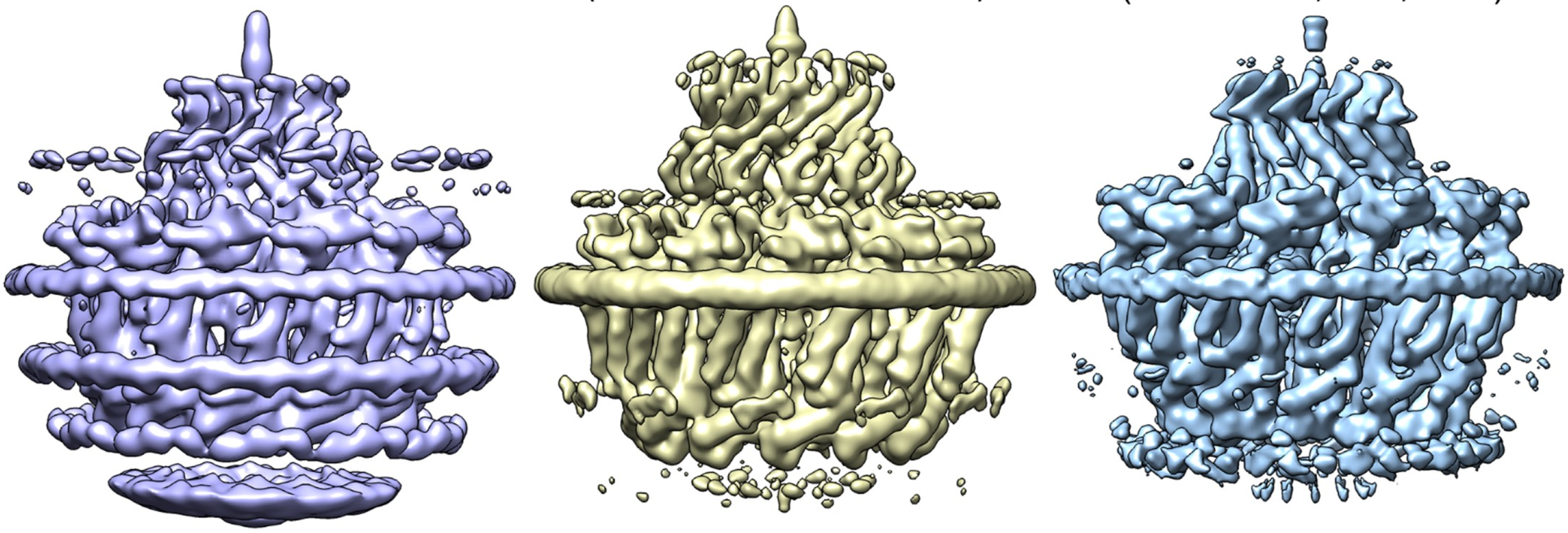

Supplementary information, Fig. S10| Portal-DNA engagement.

a The portal vertex region of the C1 capsid reconstruction of EBV, showing three layers of ringed dsDNA with strong density circling the portal inside the capsid, as indicated by the red arrows.

b Density maps of C12 portal from EBV in this study, KSHV (emd-20437) and HSV-1 (emd-9862). The three maps are all low-pass filtered to 8 Å and displayed at appropriate thresholds that show comparable portal protein densities among them.
