# Supplementary material for: CryoEM structure of the tegumented capsid of Epstein-Barr virus
Source: Cell Res. 2020 Jul 3;30(10):873–84. doi: 10.1038/s41422-020-0363-0 (PMC7608217; doi:10.1038/s41422-020-0363-0)
Supplement: Supplementary file 14 — Supplementary information, Fig. S11 [file 41422_2020_363_MOESM14_ESM.pdf]

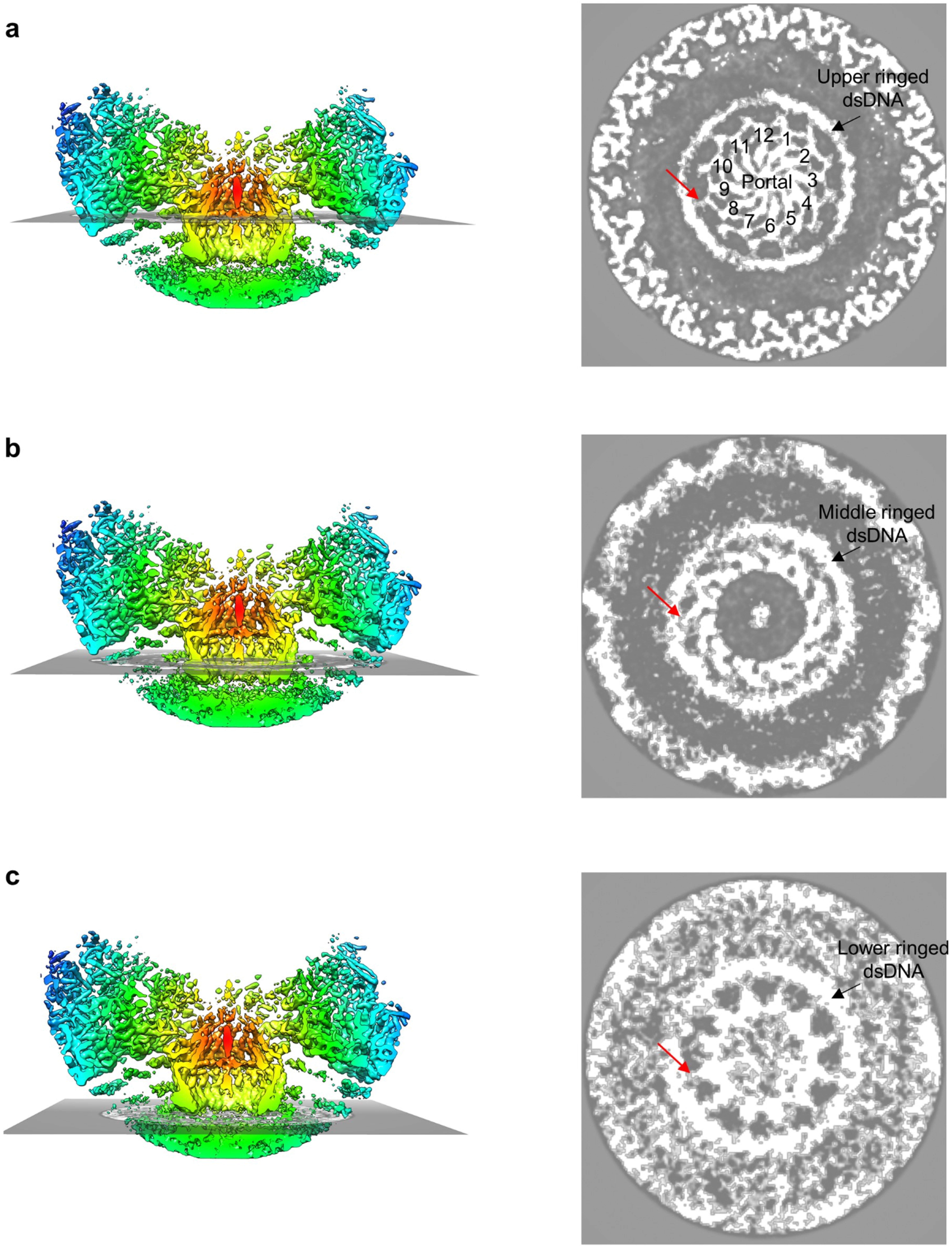

Supplementary information, Fig. S11| Interactions between portal and genome DNA.

The shaded (left) and plane (right) representations of the C1 portal vertex, showing the interactions between the portal and the upper (**a**), middle (**b**) and lower (**c**) ringed DNA, respectively. The plane representations are displayed with grayscale rendering. The red arrows indicate the connections between the portal and genomic dsDNAs.
