# Supplementary material for: CryoEM structure of the tegumented capsid of Epstein-Barr virus
Source: Cell Res. 2020 Jul 3;30(10):873–84. doi: 10.1038/s41422-020-0363-0 (PMC7608217; doi:10.1038/s41422-020-0363-0)
Supplement: Supplementary file 15 — Supplementary information, Fig. S12 [file 41422_2020_363_MOESM15_ESM.pdf]

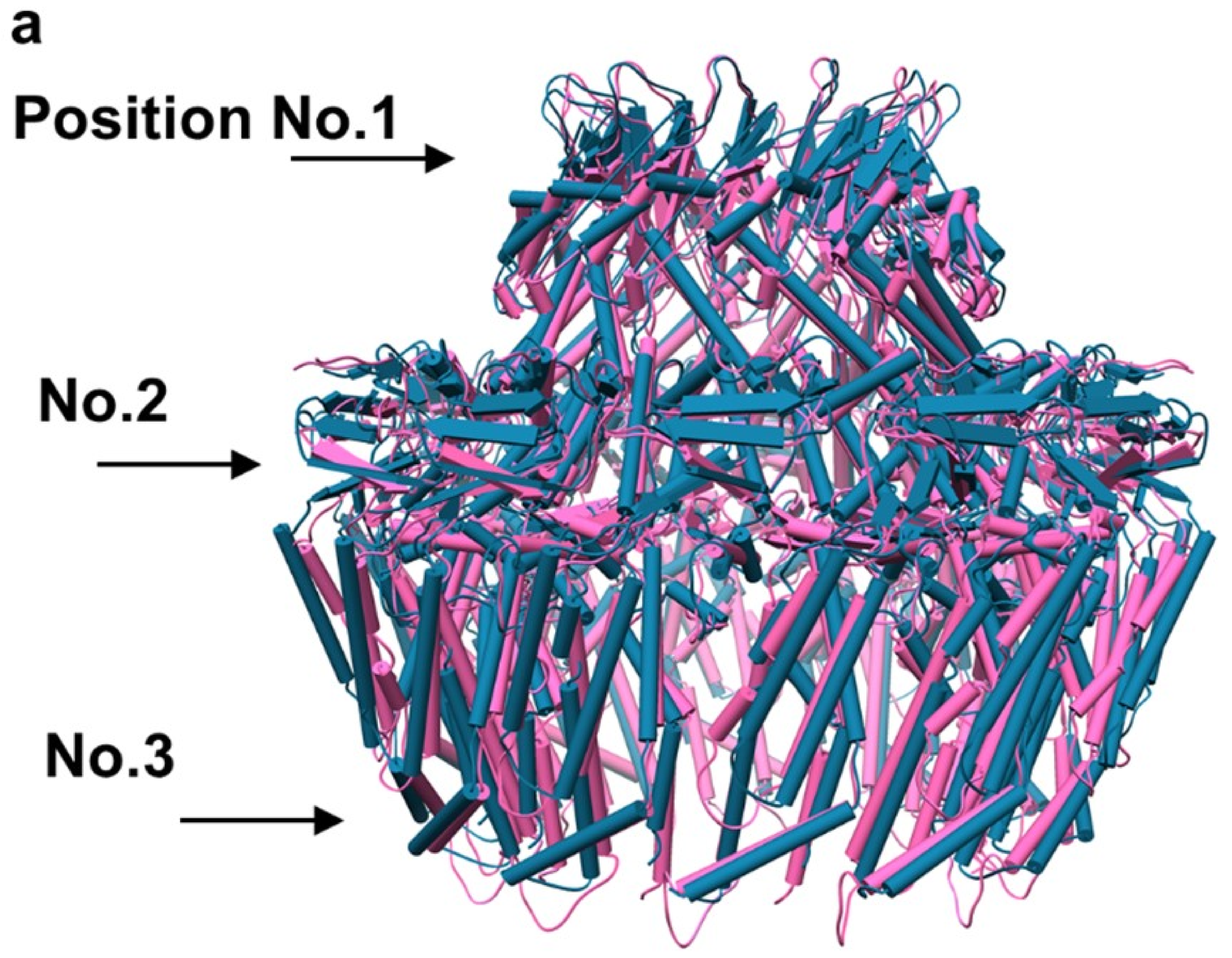

b

Position No.	*In situ*	*in vitro*-assembled (PDB: 6RVS)
1	*31*	*31*
2	*25*	*32*
3	*58*	*75*

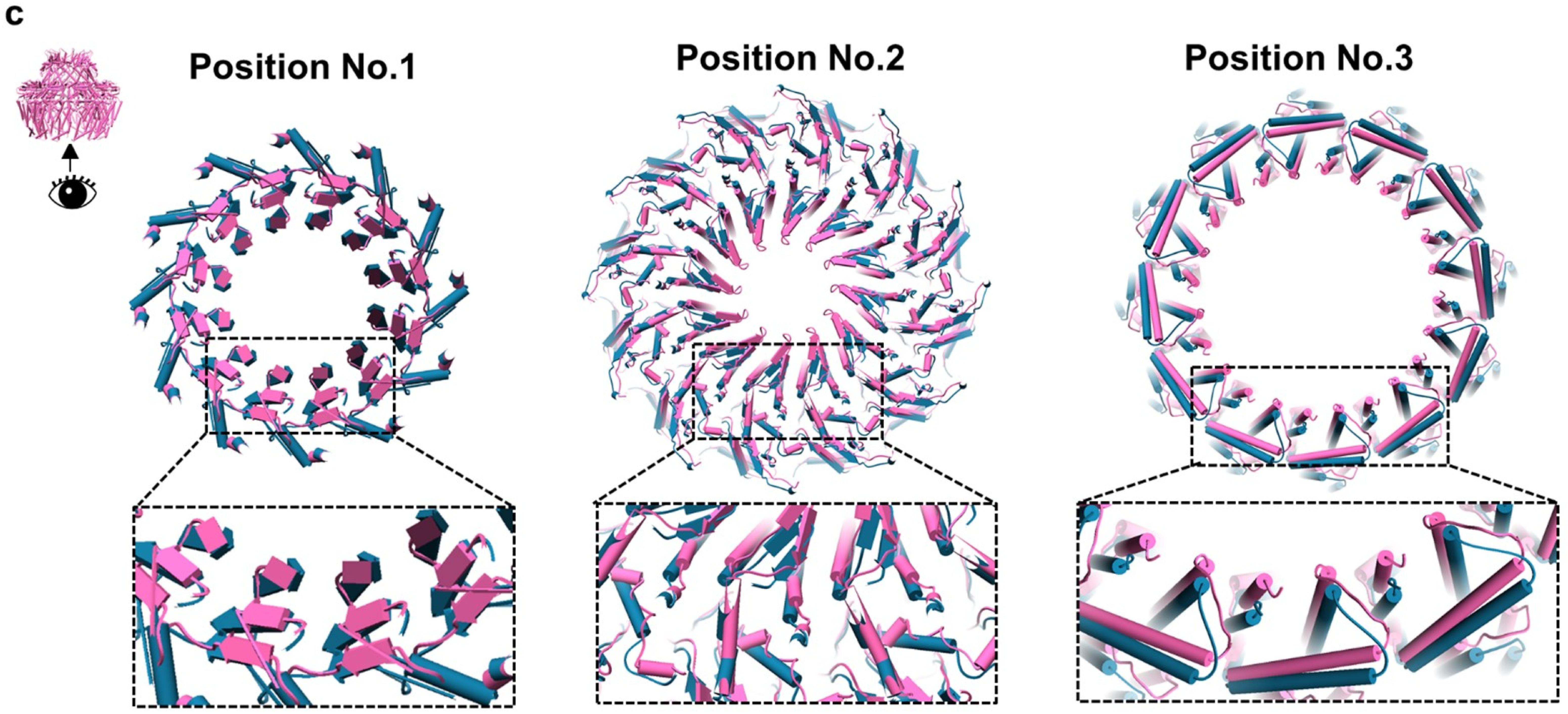

Supplementary information, Fig. S12| Structural comparison of the in situ EBV portal and the in vitro-assembled EBV portal.

a Superimposition between the *in situ* (magenta) and the *in vitro*-assembled portals (blue, PDB: 6RVS). The β-tunnel, channel valve and the most constricted region of crown domain are indicated by black arrows.

b The interior diameter comparison between the *in situ* and the *in vitro*-assembled portals at the three positions indicated in (a).

c Structural comparison between the *in situ* and the *in vitro*-assembled portals.
