# Supplementary material for: CryoEM structure of the tegumented capsid of Epstein-Barr virus
Source: Cell Res. 2020 Jul 3;30(10):873–84. doi: 10.1038/s41422-020-0363-0 (PMC7608217; doi:10.1038/s41422-020-0363-0)
Supplement: Supplementary file 16 — Supplementary information, Fig. S13 [file 41422_2020_363_MOESM16_ESM.pdf]

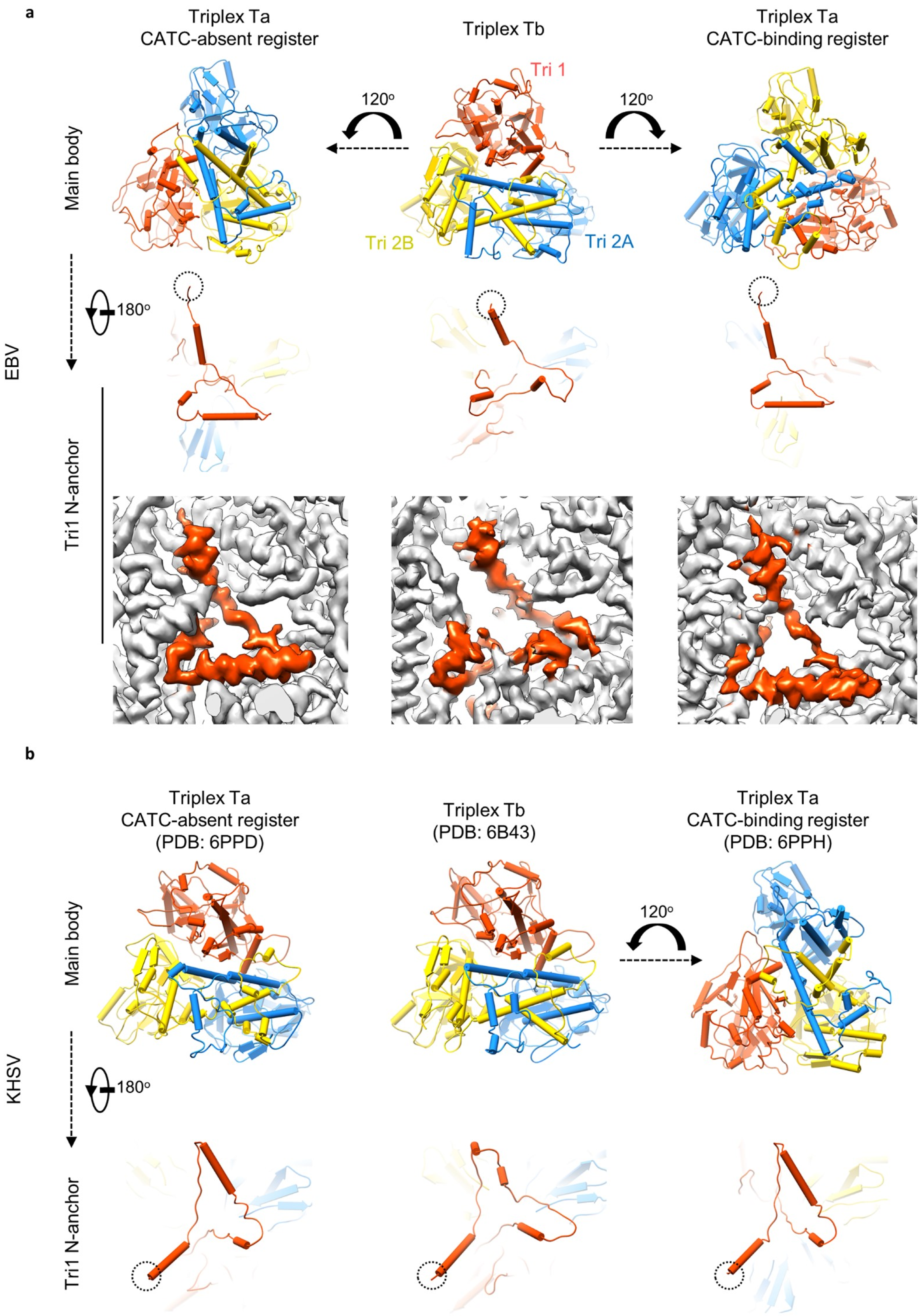

Supplementary information, Fig. S13| Structural polymorphism of triplexes of EBV and KSHV.

a Atomic models (upper and middle rows) and density maps (lower row) of EBV triplexes, showing the main bodies of both the CATC-absent (left in upper row) and CATC-binding (right in upper row) Ta rotate ~120 degree compared to that of Tb (middle in upper row) in respect to the Tri1 N-anchor (middle and lower rows).

b Atomic models of KSHV triplexes, showing the main body of only the CATC-binding (right in upper row) Ta rotate ~120 degree compared to that of Tb (middle in upper row) in respect to the Tri1 N-anchor (lower row). Since the triplexes Tb to Te adopt similar overall structural conformation, Tb is used to illustrate the structural polymorphisms of triplexes.
