# Supplementary material for: CryoEM structure of the tegumented capsid of Epstein-Barr virus
Source: Cell Res. 2020 Jul 3;30(10):873–84. doi: 10.1038/s41422-020-0363-0 (PMC7608217; doi:10.1038/s41422-020-0363-0)
Supplement: Supplementary file 17 — Supplementary information, Fig. S14 [file 41422_2020_363_MOESM17_ESM.pdf]

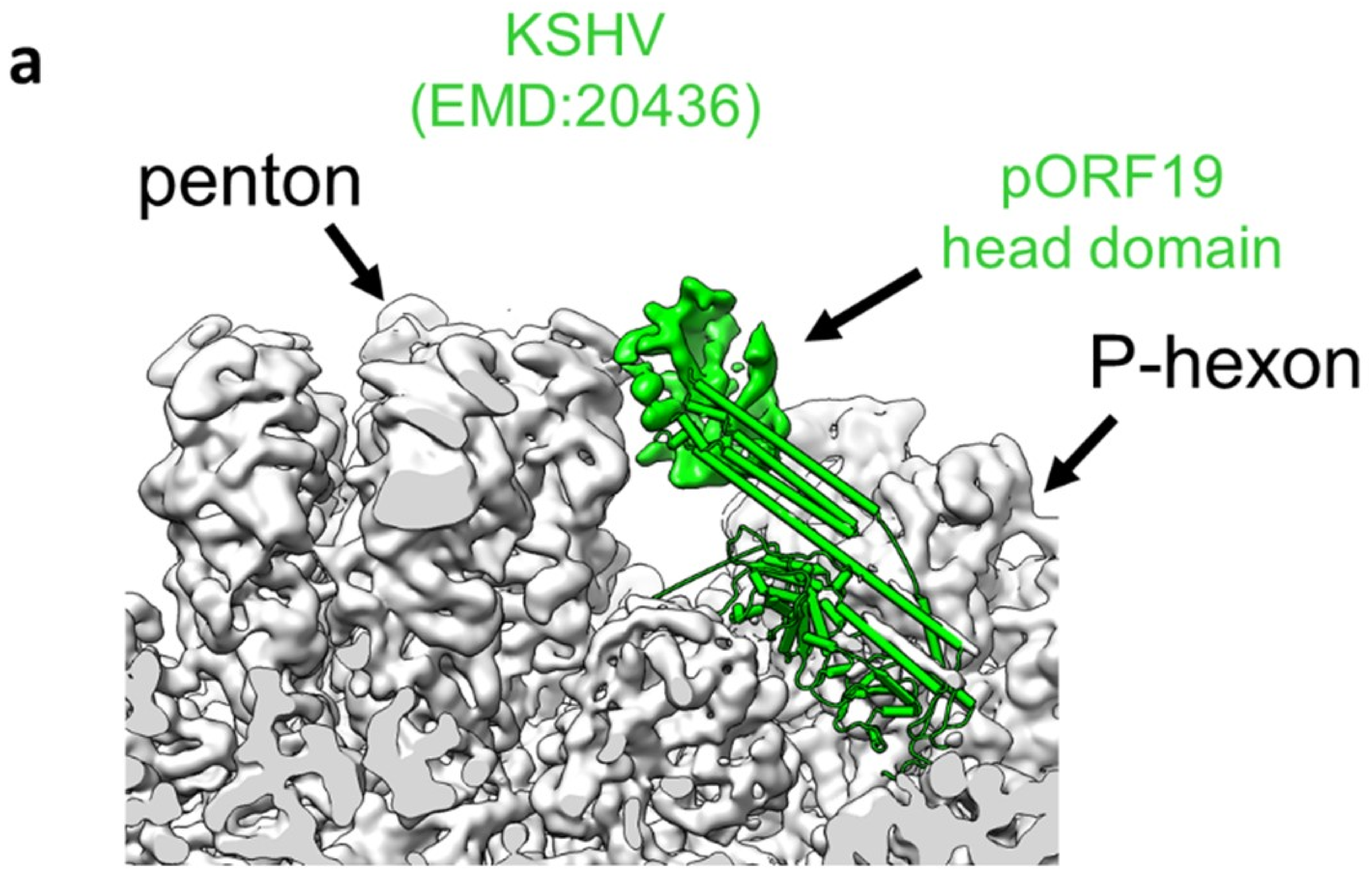

Supplementary information, Fig. S14| The interactions between CATC and penton in EBV and KSHV.

a Density map and atomic model of a selected region from the KSHV CATC-binding penton vertex, showing that the CATC of KSHV (green) interacts with only one penton MCP at its apex.

b Density map and atomic model of a selected region from the EBV portal-proximal penton vertex, showing that the CATC of EBV (red) interacts with two neighboring penton MCPs at the middle of their tower regions.

Supplementary information, Table. S1| Cryo-EM data collection, image processing, and refinement statistics

Supplementary information, Table. S2| Information of the EBV proteins involved in the formation of the capsid

Supplementary information, Movie. S1| Structures of the EBV tegumented capsid, portal vertex and dodecameric portal.
